# Supplementary material for: Health service use among adults with cerebral palsy: a mixed methods systematic review protocol
Source: BMJ Open. 2020 Aug 30;10(8):e035892. doi: 10.1136/bmjopen-2019-035892 (PMC7462157; doi:10.1136/bmjopen-2019-035892)
Supplement: Supplementary data [file bmjopen-2019-035892supp001.pdf]

Appendix 1

Medline (Ovid)

Ovid MEDLINE(R) and Epub Ahead of Print, In-Process & Other Non-Indexed Citations, Daily and Versions(R) 1946 to present

1. exp Cerebral Palsy/
2. cerebral pals$.tw.
3. ((Hemiplegi$ or diplegi$ or quadriplegi$ or unilateral$) adj5 spastic$).tw.
4. ((Hemiplegi$ or diplegi$ or quadriplegi$ or unilateral$) adj3 ataxi$).tw.
5. Little$ disease.tw.
6. 1 or 2 or 3 or 4 or 5

7. exp Health Services/
8. exp Health Facilities/
9. exp Comprehensive Health Care/
10. exp "Delivery of Health Care"/
11. exp "analytical, diagnostic and therapeutic techniques and equipment (non mesh)"/
12. exp residential treatment/
13. emergenc$.tw.
14. therap$.tw.
15. diagnos$.tw.
16. assistive device$.tw.

17. health service$.tw.
18. health care$.tw.
19. hospital$.tw.
20. rehabilitation$.tw.
21. general practice$.tw.
22. family practice$.tw.
23. primary care$.tw.
24. community$.tw.
25. respite$.tw.
26. residential care$.tw
27. 7 or 8 or 9 or 10 or 11 or 12 or 13 or 14 or 15 or 16 or 17 or 18 or 19 or 20 or 21 or 22 or 23 or 24 or 25 or 26
28. exp Adult/
29. adult$.tw.
30. elder$.tw.
31. older$.tw.
32. young adult$.tw.
33. 28 or 29 or 30 or 31 or 32
34. 6 AND 27 AND 33
