# Supplementary material for: Health service use among adults with cerebral palsy: a mixed methods systematic review protocol
Source: BMJ Open. 2020 Aug 30;10(8):e035892. doi: 10.1136/bmjopen-2019-035892 (PMC7462157; doi:10.1136/bmjopen-2019-035892)
Supplement: Author's manuscript [file bmjopen-2019-035892.draft_revisions.pdf]

BMJ Open is committed to open peer review. As part of this commitment we make the peer review history of every article we publish publicly available.

When an article is published we post the peer reviewers' comments and the authors' responses online. We also post the versions of the paper that were used during peer review. These are the versions that the peer review comments apply to.

The versions of the paper that follow are the versions that were submitted during the peer review process. They are not the versions of record or the final published versions. They should not be cited or distributed as the published version of this manuscript.

BMJ Open is an open access journal and the full, final, typeset and author-corrected version of record of the manuscript is available on our site with no access controls, subscription charges or pay-per-view fees (http://bmjopen.bmj.com).

If you have any questions on BMJ Open's open peer review process please email info.bmjopen@bmj.com

BMJ Open

A MIXED METHODS SYSTEMATIC REVIEW PROTOCOL OF HEALTH SERVICE USE AMONG ADULTS WITH CEREBRAL PALSY

Journal:	*BMJ Open*
Manuscript ID	bmjopen-2019-035892
Article Type:	Protocol
Date Submitted by the Author:	20-Nov-2019

Complete List of Authors:	Manikandan, Manjula; Royal College of Surgeons in Ireland, Department of Public Health and Epidemiology<br>Walsh, Aisling; Royal College of Surgeons in Ireland, Department of Public Health and Epidemiology<br>Kerr, C ; Queen's University Belfast, School of Nursing and Midwifery<br>Walsh, Michael; Health Service Executive, Office of the Chief Clinical Officer<br>Ryan, Jennifer; Brunel University London, College of Health and Life Sciences; Royal College of Surgeons in Ireland, Department of Public Health and Epidemiology

Keywords:	Adult neurology < NEUROLOGY, REHABILITATION MEDICINE, HEALTH SERVICES ADMINISTRATION & MANAGEMENT

SCHOLARONE™
Manuscripts

I, the Submitting Author has the right to grant and does grant on behalf of all authors of the Work (as defined in the below author licence), an exclusive licence and/or a non-exclusive licence for contributions from authors who are: i) UK Crown employees; ii) where BMJ has agreed a CC-BY licence shall apply, and/or iii) in accordance with the terms applicable for US Federal Government officers or employees acting as part of their official duties; on a worldwide, perpetual, irrevocable, royalty-free basis to BMJ Publishing Group Ltd ("BMJ") its licensees and where the relevant Journal is co-owned by BMJ to the co-owners of the Journal, to publish the Work in this journal and any other BMJ products and to exploit all rights, as set out in our licence.

The Submitting Author accepts and understands that any supply made under these terms is made by BMJ to the Submitting Author unless you are acting as an employee on behalf of your employer or a postgraduate student of an affiliated institution which is paying any applicable article publishing charge ("APC") for Open Access articles. Where the Submitting Author wishes to make the Work available on an Open Access basis (and intends to pay the relevant APC), the terms of reuse of such Open Access shall be governed by a Creative Commons licence – details of these licences and which Creative Commons licence will apply to this Work are set out in our licence referred to above.

Other than as permitted in any relevant BMJ Author's Self Archiving Policies, I confirm this Work has not been accepted for publication elsewhere, is not being considered for publication elsewhere and does not duplicate material already published. I confirm all authors consent to publication of this Work and authorise the granting of this licence.

For peer review only

A MIXED METHODS SYSTEMATIC REVIEW PROTOCOL OF HEALTH SERVICE USE AMONG ADULTS WITH CEREBRAL PALSY

Manjula Manikandan[1], Aisling Walsh[1], Claire Kerr[2], Michael Walsh[4], Jennifer Ryan[1, 3]

[1]Department of Public Health and Epidemiology, Royal College of Surgeons in Ireland, Ireland

[2]School of Nursing and Midwifery, Queen's University Belfast, United Kingdom

[3]College of Health and Life Sciences, Brunel University London, United Kingdom

[4]Office of the Chief Clinical Officer, Health Service Executive, Ireland

Email: manjulamanikandan@rcsi.ie, aislingwalsh@rcsi.ie, c.kerr@qub.ac.uk, michael.walsh14@hse.ie, jenniferryan@rcsi.ie

Corresponding author: Jennifer Ryan and Manjula Manikandan; RCSI Department of Public Health and Epidemiology, Royal College of Surgeons in Ireland, Lower Mercer Street, Dublin 2, Ireland.

Telephone number of the corresponding author: +353 1402 2413; +353 1402 8507

Keywords: adults; cerebral palsy; health service use; experiences

Word count: 2436 words (excluding references)

Abstract word count: 309 words

For peer review only - http://bmjopen.bmj.com/site/about/guidelines.xhtml

ABSTRACT

Introduction: Cerebral palsy (CP) is a childhood neurodisability that primarily results in motor impairments and activity limitations, but is often associated with epilepsy and disturbances of sensation, perception, cognition, behaviour and speech. Most children with CP survive well into adulthood. As adults, people with CP experience secondary conditions in addition to the ongoing health issues from childhood. Therefore, adults with CP often require extensive health services. However, health service use among adults with CP remains unclear. This mixed method review aims to identify, appraise and synthesise the available quantitative and qualitative literature examining health service use among adults with CP.

Methods and analysis: The mixed method systematic review will be conducted in accordance with the Joanna Briggs Institute (JBI) methodology. A systematic search of Medline (Ovid), CINAHL, EMBASE, PsychINFO and Cochrane library from inception will be conducted. Quantitative observational studies, qualitative studies, and mixed method studies examining health service use among adults with CP (≥ 18 years) will be included. Outcomes of interest are the proportion of adults using health services, the frequency of health service use and the experiences and perceptions of health services for adults with CP. Two reviewers will independently screen titles, abstracts and full texts, extract data, and assess the quality of included studies using instruments from the JBI. Where possible a meta-analysis and meta aggregation of findings will be performed for quantitative and qualitative data, respectively. The quantitative and qualitative findings will be integrated using a triangulation approach at the synthesis stage. A narrative synthesis will be carried out where this is not possible.

Ethics and dissemination: Ethics approval is not required for this review. The findings will be disseminated through a peer-reviewed journal, conferences for researchers, health professionals and people with CP, and charity and voluntary agencies that support people with CP.

For peer review only - http://bmjopen.bmj.com/site/about/guidelines.xhtml

Systematic review registration number: The protocol is under review at PROSPERO.

STRENGTHS & LIMITATIONS OF THIS STUDY

- This mixed method systematic review will include quantitative, qualitative and mixed methods studies in order to provide a comprehensive overview of the current literature relating to health services for adults with cerebral palsy.
- This review will use the Andersen-Newman behavioural model of health service utilisation as an overarching framework to guide data extraction and analysis.
- Only studies that are published in English will be included in the review.
- Heterogeneity of eligible studies may limit the possibility of meta-analysis.

BACKGROUND

Cerebral palsy (CP) is a "group of permanent disorders of movement and posture, causing activity limitation, that are linked to non-progressive disturbances of the developing fetal or infant brain"[1]. To be diagnosed as someone with CP, the disturbance to the brain occurs before, during or up to approximately 2 years after birth [1]. People with CP present with different types of motor abnormality, namely spasticity, dyskinesia and ataxia [1]. In addition to movement disorders, people with CP may experience epilepsy or impairments of cognition, speech, hearing and vision [1].

Traditionally, CP was considered a paediatric illness, however children with CP survive well-into adulthood. A recent study reports more than 80% of people with CP have a life expectancy, beyond 58 years [2]. The incidence rate of CP remains steady between 2.0-2.5 cases per 1000 live births, however the life expectancy has increased and it suggests that the prevalence of CP is increasing [3, 4]. As adults, people with CP continue to present with ongoing health issues from childhood, in addition to the issues related to ageing. For instance, adults with CP experience increased risk of arthritis, stroke, heart failure, ischaemic heart disease, chronic respiratory disease and mental health disorders compared to adults

For peer review only - http://bmjopen.bmj.com/site/about/guidelines.xhtml

without CP [5-8]. Studies have also reported increased fatigue, pain and a decline in mobility, as people with CP move into adulthood [9]. Therefore, adults with CP may require extensive health services.

The National Institute for Health and Care Excellence (NICE) guideline for cerebral palsy in adults has recommended continuity of care for adults with CP, tailored to their needs [10]. This continuity of care is likely to avoid emergency hospital admissions[10]. However, studies of adolescents with CP have reported challenging experiences in navigating services during transition from child to adult services [11, 12]. In addition, adolescents with CP reported anxiety about transfer from child to adult services as they perceived that services in adulthood were poorly co-ordinated and managed [11-13]. As the evidence describing the long-term health issues experienced by adults with CP has expanded in recent years, there is a growing need for research examining health service use among adults with CP.

'Health services' are defined as those "services that are first and foremost undertaken to have direct effect on people's health. These extend from health promotion and disease prevention, through curative services, to long term care and rehabilitation"[14]. Use of health services may be associated with various factors relating to the individual and their environment [15]. The Andersen-Newman behavioural model of health service utilisation (ANM) describes environmental factors, population factors and outcomes that can be associated with health service use behaviour [15, 16]. Environmental factors include those relating to the health care system (e.g., national policies, resources and organisations involved) and external environment (e.g., physical, political and economic factors) [16]. Population factors include demographics (e.g. age, gender, ethnicity, location and condition-specific characteristics such as functional mobility and type of motor abnormality) [16]. Outcomes include perceived and evaluated health status and customer satisfaction [16].

For peer review only - http://bmjopen.bmj.com/site/about/guidelines.xhtml

A preliminary search of PROSPERO, MEDLINE (Ovid) and the Cochrane Database of Systematic Reviews indicated that no systematic reviews have been conducted examining health service use among adults with CP. However, such a review would potentially inform health service used by adults with CP, direct research efforts, inform service delivery and identify areas of challenges faced by adults with CP. Therefore, the aim of this mixed method systematic review is to identify, appraise and synthesise the available quantitative and qualitative literature examining health service use among adults with CP.

For peer review only

OBJECTIVES

The objectives are:

1. To investigate the proportion of adults using health services and the frequency of health service use among adults with CP.
2. To examine associations between environmental factors, population factors and outcomes as described by the ANM, and health service use among adults with CP.
3. To explore the experiences and perceptions of health services for adults with CP, from the perspective of adults with CP, caregivers and health professionals.

METHODS

This mixed method systematic review will be conducted in accordance with the Joanna Briggs Institute methodology guidelines [17]. The protocol is under review for registration on the PROSPERO database of systematic reviews and is written according to PRISMA-P checklist [18]. The review will be reported using MOOSE and ENTREQ reporting guidelines [19, 20].

Study eligibility

Study design

For peer review only - http://bmjopen.bmj.com/site/about/guidelines.xhtml

The review will include quantitative, qualitative and mixed method studies. Quantitative studies will include observational studies (e.g., cross-sectional, case-control and cohort studies). Qualitative studies will include studies of recognised qualitative approaches (e.g., interviews, focus groups, observation, thematic analysis, content analysis, narrative analysis and framework analysis) [21]. Mixed method studies will only be considered if quantitative or qualitative components can be clearly extracted.

Studies published in English language will be included. However, there will be no restriction on the geographic location of studies published.

Participants

Studies of adults with CP (≥ 18 years) with all types of motor disorders and all levels of functional mobility will be included. The functional mobility for people with CP is widely classified using the Gross Motor Function Classification System (GMFCS) [22]. For qualitative studies, studies including caregivers and health professionals will also be included. For studies that include children and young adults (for example, those aged 15-25 years), data from people ≥18 years only will be included, if possible. If it is not possible to extract data for adults only, we will include data if the mean age of the sample is ≥18 years. Where studies include data on adults with CP as part of a larger sample of people with disabilities, we will extract data on adults with CP only, if possible.

Outcome

Health services for this review will include services that have direct impact on adults with CP's health [14, 23-26]. This can include hospital admissions, emergency department visits, outpatient visits to medical and allied health professionals (e.g. physicians, pharmacist, physiotherapists, occupational therapists, podiatrists, psychologists, orthotists, speech and language therapists). In addition, diagnostic or assistive device services (e.g. radiologists, communication aids, mobility aids and wheelchair services) and support

For peer review only - http://bmjopen.bmj.com/site/about/guidelines.xhtml

services (e.g. supported living, personal assistance, residential, respite and domiciliary services) will also be included [14, 23-26].

For the quantitative component of this review, the proportion of adults using health services and the frequency of health service use will be the main outcomes. Where possible, we will report each type of health services used by adults with CP.

For the qualitative component, the main outcomes are experience and perceptions of using health services among adults with CP. We will also explore experiences and perceptions of health services for adults with CP from the perspectives of caregivers, and health professionals.

Exclusion criteria

We will exclude reviews, dissertations, editorials, commentaries and conference abstracts. Studies of children and young people with CP (<18 years) will be excluded. Studies looking at services that have an indirect effect on people's health only such as education, employment, housing and transport will be excluded [14]. Finally, randomised controlled studies on intervention effectiveness will be excluded from this review.

Search Strategy

The electronic databases Medline (Ovid), CINAHL, EMBASE, PsychINFO and Cochrane library will be systematically searched from inception of each database to November 2019. We will conduct a pilot search in Medline (Ovid) to inform the search strategy. A librarian will support development of the search strategy. Reference lists of included studies will be screened.

Search terms

The search strategy will consist of fully exploded subject headings and free text words relating to adults with CP and health services. The search strategy developed from Medline (Ovid) will be tailored to each

For peer review only - http://bmjopen.bmj.com/site/about/guidelines.xhtml

database. No filters on study design or settings will be applied at this stage. Search terms will be combined using Boolean operators. A sample search strategy for Medline (Ovid) is provided in Appendix 1.

Data management

A search log will be maintained to record the databases, keywords used and results of each search. The titles and abstracts of papers identified from the systematic search will be imported into reference management software (Endnote X8.2) and duplicates will be removed.

Study selection

Two reviewers will screen titles and abstracts independently. Where studies meet the inclusion criteria or it is unclear if they are eligible, full-texts will be retrieved for further assessment. The two reviewers will screen full text articles for inclusion independently. Conflicts will be resolved by discussion or third reviewer involvement. The summary of the search results will be presented in a Preferred Reporting Items for Systematic Reviews and Meta-analyses (PRISMA) flow diagram [27].

Data extraction

Two reviewers will extract data independently and any disagreements will be resolved initially through discussion and if required, discussion with a third reviewer. The study authors will be contacted to retrieve missing data necessary for meta-analysis or meta-aggregation.

Data items

A data extraction form will be developed and piloted by two reviewers prior to data extraction. For quantitative studies and quantitative components of mixed method studies, extracted data will include data on study design, country, participant characteristics, proportion of people using each health service,

For peer review only - http://bmjopen.bmj.com/site/about/guidelines.xhtml

frequency of use of each health service, and variables relating to the environmental factors, population factors and outcomes according to the ANM. For qualitative studies and the qualitative components of mixed method studies, the extracted data will include data on study design, setting, country, methodology, participants, data analysis and themes related to experiences. The qualitative data, such as quotes or themes of experiences and perceptions will be extracted from the results sections of the included studies.

Quality assessment

Two reviewers will independently assess the methodological quality of included studies using standardised critical appraisal instruments from the Joanna Briggs Institute for qualitative and quantitative studies [17]. Disagreements between the reviewers will be discussed and discrepancies resolved by a third reviewer. The results of the quality appraisal will be reported in a table and narrative form.

Data synthesis

Quantitative studies

The proportion of people using health services and frequency of health service use will be pooled according to each type of health service. We will standardise frequency to a specific time frame. Separate meta-analyses will be conducted for each service. We will use a fixed-effects model. We will assess statistical heterogeneity using the I^2 statistic and a Chi^2 test [28]. If data allow, we will explore factors associated with health service use using meta-regression. If it is not possible to perform a meta-regression, sub-group analyses may be conducted according to characteristics potentially associated with health service use as outlined in the ANM. Where data cannot be pooled, a narrative synthesis of the quantitative findings will be conducted.

For peer review only - http://bmjopen.bmj.com/site/about/guidelines.xhtml

For peer review only

Qualitative studies

Qualitative experiences from different perspectives will be pooled using a meta-aggregation approach [29]. This involves identifying the findings, categorising the findings based on similarity in meaning and grouping the categories into synthesised findings [29]. Where textual-pooling is not possible, the findings will be presented in narrative form.

Integration

The findings from quantitative and qualitative synthesis will be integrated using a convergent segregated approach, to preserve the integrity of quantitative and qualitative findings [30]. The results from quantitative and qualitative components will be triangulated and reported using the ANM as a framework. Triangulation will involve comparing or organising quantitative and qualitative findings into a line of argument via tabulation, groupings and visual representation to produce an overall configured analysis [31]. A narrative form of discussion will be considered, where this is not possible.

ETHICS AND DISSEMINATION

Ethical approval is not required for this review as both quantitative and qualitative data used for analysis will be extracted from published studies. The review will be published in a peer-reviewed journal and presented at conferences for researchers, health professionals and people with CP. Findings will be disseminated to healthcare providers and shared with charity and voluntary agencies that support people with CP.

DISCUSSION

The aim of this mixed method systematic review is to identify, appraise and synthesise the available quantitative and qualitative literature examining health service use among adults with CP. The specific

For peer review only - http://bmjopen.bmj.com/site/about/guidelines.xhtml

For peer review only

objectives are (1) to investigate the proportion of adults using health services and the frequency of health service use among adults with CP, (2) to examine associations between environmental factors, population factors and outcomes as described by the ANM, and health service use among adults with CP, and (3) to explore the experiences of health services for adults with CP, from the perspective of adults with CP, caregivers and health professionals.

The majority of people with CP will survive to at least 60 years of age [2]. Many adults with CP experience secondary conditions with age that require intervention and support from clinicians working across multiple health and social care disciplines. Previous research has demonstrated that young people with CP experience challenges in accessing health services, as they move from child to adult health services [11, 13, 32-34]. A review of health service use among adults with CP is required in order to direct research efforts and inform service provision. It is anticipated that this mixed methods review will provide greater insights into the health services used by adults with CP.

Limitations of this review are exclusion of studies that are not peer-reviewed or published in languages other than English, potentially resulting in less generalisable findings. Finally, it may not be possible to conduct a meta-analysis due to a lack of data and/or heterogeneity.

AUTHOR CONTRIBUTIONS

All authors have agreed on the final version and meet at least two of the following criteria (recommended by ICMJE www.icmje.org/recommendations):

1. Substantial contributions to the conception or design of the work; or the acquisition, analysis, or interpretation of data for the work **(MM, AW, CK, MW and JR)**
2. Drafting the work or revising it critically for important intellectual content (**MM, JR)**
3. Final approval of the version to be published **(MM, AW, CK, MW and JR)**

For peer review only - http://bmjopen.bmj.com/site/about/guidelines.xhtml

4. Agreement to be accountable for all aspects of the work in ensuring that questions related to the accuracy or integrity of any part of the work are appropriately investigated and resolved **(MM and JR).**

ACKNOWLEDGEMENTS

The authors acknowledge the support from the RCSI librarian (Ms Grainne McCabe) for supporting the development of search terms with the reviewers.

COMPETING INTERESTS

The authors disclose no competing interests.

FUNDING

This work was conducted as part of the SPHeRE Programme under Grant No. SPHeRE/2013/1. This review is funded by the Royal College of Surgeons in Ireland (RCSI) through the StAR programme. The funders had no role in developing the protocol.

REFERENCES

1. Colver A, Fairhurst C, Pharoah PO. Cerebral palsy. Lancet (London, England). 2014;383(9924):1240-9.
2. Blair E, Langdon K, McIntyre S, Lawrence D, Watson L. Survival and mortality in cerebral palsy: observations to the sixth decade from a data linkage study of a total population register and National Death Index. BMC neurology. 2019;19(1):111.
3. Johnson A. Prevalence and characteristics of children with cerebral palsy in Europe. Developmental Medicine & Child Neurology. 2002;44(9):633-40.
4. Oskoui M, Coutinho F, Dykeman J, Jette N, Pringsheim T. An update on the prevalence of cerebral palsy: a systematic review and meta-analysis. Developmental medicine and child neurology. 2013;55(6):509-19.
5. Peterson MD, Ryan JM, Hurvitz EA, Mahmoudi E. Chronic Conditions in Adults With Cerebral Palsy. Jama. 2015;314(21):2303-5.
6. Ryan JM, Peterson MD, Matthews A, Ryan N, Smith KJ, O'Connell NE, et al. Noncommunicable disease among adults with cerebral palsy: A matched cohort study. Neurology. 2019;93(14):e1385-e96.
7. Smith KJ, Peterson MD, O'Connell NE, Victor C, Liverani S, Anokye N, et al. Risk of depression and anxiety in adults with cerebral palsy. JAMA neurology. 2019;76(3):294-300.
8. O'Connell NE, Smith KJ, Peterson MD, Ryan N, Liverani S, Anokye N, et al. Incidence of osteoarthritis, osteoporosis and inflammatory musculoskeletal diseases in adults with cerebral palsy: A population-based cohort study. Bone. 2019;125:30-5.

For peer review only - http://bmjopen.bmj.com/site/about/guidelines.xhtml

9. Bottos M, Feliciangeli A, Sciuto L, Gericke C, Vianello A. Functional status of adults with cerebral palsy and implications for treatment of children. Developmental Medicine & Child Neurology. 2001;43(8):516-28.
10. National Institute for Health and Care Excellence (NICE). Cerebral Palsy in adults London 2019 [Available from: https://www.nice.org.uk/guidance/ng119/chapter/Rationale-and-impact.
11. Hislop J, Mason H, Parr JR, Vale L, Colver A. Views of Young People With Chronic Conditions on Transition From Pediatric to Adult Health Services. Journal of Adolescent Health. 2016;59(3):345-53.
12. Wright AE, Robb J, Shearer MC. Transition from paediatric to adult health services in Scotland for young people with cerebral palsy. Journal of child health care : for professionals working with children in the hospital and community. 2016;20(2):205-13.
13. Colver A, Pearse R, Watson RM, Fay M, Rapley T, Mann KD, et al. How well do services for young people with long term conditions deliver features proposed to improve transition? BMC Health Serv Res. 2018;18(1):337.
14. Black N, Gruen R. Understanding health services: McGraw-Hill Education (UK); 2005.
15. Andersen RM. Revisiting the behavioral model and access to medical care: does it matter? Journal of health and social behavior. 1995:1-10.
16. Andersen R, Newman JF. Societal and individual determinants of medical care utilization in the United States. The Milbank Memorial Fund Quarterly Health and Society. 1973:95-124.
17. The Joanna Briggs Institute. Joanna Briggs Institute Reviewer's Manual. University of Adelaide, South Australia2017. Available from: https://wiki.joannabriggs.org/display/MANUAL/About+this+Manual.
18. Moher D, Shamseer L, Clarke M, Ghersi D, Liberati A, Petticrew M, et al. Preferred reporting items for systematic review and meta-analysis protocols (PRISMA-P) 2015 statement. Systematic reviews. 2015;4(1):1.
19. Tong A, Flemming K, McInnes E, Oliver S, Craig J. Enhancing transparency in reporting the synthesis of qualitative research: ENTREQ. BMC medical research methodology. 2012;12:181.
20. Stroup DF, Berlin JA, Morton SC, Olkin I, Williamson GD, Rennie D, et al. Meta-analysis of observational studies in epidemiology: a proposal for reporting. Meta-analysis Of Observational Studies in Epidemiology (MOOSE) group. Jama. 2000;283(15):2008-12.
21. Creswell JW, Poth CN. Qualitative inquiry and research design: Choosing among five approaches. 4th ed: Sage publications; 2017.
22. Palisano R, Rosenbaum P, Walter S, Russell D, Wood E, Galuppi B. Development and reliability of a system to classify gross motor function in children with cerebral palsy. Developmental medicine and child neurology. 1997;39(4):214-23.
23. Cheng SM, Catallo C. Case definition for health and social care services integrated initiatives. Journal of Integrated Care. 2019.
24. Leutz WN. Five laws for integrating medical and social services: lessons from the United States and the United Kingdom. The Milbank Quarterly. 1999;77(1):77-110.
25. Leichsenring K. Developing integrated health and social care services for older persons in Europe. Int J Integr Care. 2004;4:e10-e.
26. Dickens J. The definition of social work in the United Kingdom, 2000–2010. International Journal of Social Welfare. 2012;21(1):34-43.
27. Moher D, Liberati A, Tetzlaff J, Altman DG. Preferred reporting items for systematic reviews and meta-analyses: the PRISMA statement. Annals of internal medicine. 2009;151(4):264-9.
28. Munn Z, Tufanaru C, Aromataris E. JBI's systematic reviews: data extraction and synthesis. AJN The American Journal of Nursing. 2014;114(7):49-54.
29. Pearson A. Balancing the evidence: incorporating the synthesis of qualitative data into systematic reviews. JBI reports. 2004;2(2):45-64.

For peer review only - http://bmjopen.bmj.com/site/about/guidelines.xhtml

30. Joanna Briggs Institute Reviewers'. Methodology for JBI Mixed Methods Systematic Reviews 2014 [Available from: https://bit.ly/2JWOUsL.
31. Noyes J, Booth A, Moore G, Flemming K, Tunçalp Ö, Shakibazadeh E. Synthesising quantitative and qualitative evidence to inform guidelines on complex interventions: clarifying the purposes, designs and outlining some methods. BMJ global health. 2019;4(Suppl 1):e000893.
32. Colver A, McConachie H, Le Couteur A, Dovey-Pearce G, Mann K, McDonagh J, et al. A longitudinal, observational study of the features of transitional healthcare associated with better outcomes for young people with long-term conditions. BMC medicine. 2018;16(1):111.
33. Bagatell N, Chan D, Rauch KK, Thorpe D. "Thrust into adulthood": Transition experiences of young adults with cerebral palsy. Disability and Health Journal. 2017;10(1):80-6.
34. Kolehmainen N, McCafferty S, Maniatopoulos G, Vale L, Le-Couteur AS, Colver A. What constitutes successful commissioning of transition from children's to adults' services for young people with long-term conditions and what are the challenges? An interview study. BMJ Paediatrics Open. 2017;1(1).

For peer review only

For peer review only - http://bmjopen.bmj.com/site/about/guidelines.xhtml

Appendix 1

Medline (Ovid)

Ovid MEDLINE(R) and Epub Ahead of Print, In-Process & Other Non-Indexed Citations, Daily and Versions(R) 1946 to present

1. exp Cerebral Palsy/
2. cerebral pals$.tw.
3. ((Hemiplegi$ or diplegi$ or quadriplegi$ or unilateral$) adj5 spastic$).tw.
4. ((Hemiplegi$ or diplegi$ or quadriplegi$ or unilateral$) adj3 ataxi$).tw.
5. Little$ disease.tw.
6. 1 or 2 or 3 or 4 or 5

7. exp Health Services/
8. exp Health Facilities/
9. exp Comprehensive Health Care/
10. exp "Delivery of Health Care"/
11. exp "analytical, diagnostic and therapeutic techniques and equipment (non mesh)"/
12. exp residential treatment/
13. emergenc$.tw.
14. therap$.tw.
15. diagnos$.tw.
16. assistive device$.tw.

For peer review only

17. health service$.tw.

18. health care$.tw.

19. hospital$.tw.

20. rehabilitation$.tw.

21. general practice$.tw.

22. family practice$.tw.

23. primary care$.tw.

24. community$.tw.

25. respite$.tw.

26. residential care$.tw

27. 7 or 8 or 9 or 10 or 11 or 12 or 13 or 14 or 15 or 16 or 17 or 18 or 19 or 20 or 21 or 22 or 23 or 24 or 25 or 26

28. exp Adult/

29. adult$.tw.

30. elder$.tw.

31. older$.tw.

32. young adult$.tw.

33. 28 or 29 or 30 or 31 or 32

34. 6 AND 27 AND 33

For peer review only

Reporting checklist for protocol of a systematic review.

Based on the PRISMA-P guidelines.

Instructions to authors

Complete this checklist by entering the page numbers from your manuscript where readers will find each of the items listed below.

Your article may not currently address all the items on the checklist. Please modify your text to include the missing information. If you are certain that an item does not apply, please write "n/a" and provide a short explanation.

Upload your completed checklist as an extra file when you submit to a journal.

In your methods section, say that you used the PRISMA-Preporting guidelines, and cite them as:

Moher D, Shamseer L, Clarke M, Ghersi D, Liberati A, Petticrew M, Shekelle P, Stewart LA. Preferred Reporting Items for Systematic Review and Meta-Analysis Protocols (PRISMA-P) 2015 statement. Syst Rev. 2015;4(1):1.

For peer review only

		Reporting Item	Page Number
**Title**			
Identification	#1a	Identify the report as a protocol of a systematic review	1
Update	#1b	If the protocol is for an update of a previous systematic review, identify as such	n/a

**Registration**			
	#2	If registered, provide the name of the registry (such as PROSPERO) and registration number	3
**Authors**			
Contact	#3a	Provide name, institutional affiliation, e-mail address of all protocol authors; provide physical mailing address of corresponding author	1
Contribution	#3b	Describe contributions of protocol authors and identify the guarantor of the review	11,12
**Amendments**			
	#4	If the protocol represents an amendment of a previously completed or published protocol, identify as such and list changes; otherwise, state plan for documenting important protocol amendments	n/a
**Support**			
Sources	#5a	Indicate sources of financial or other support for the review	12
Sponsor	#5b	Provide name for the review funder and / or sponsor	12
Role of sponsor or funder	#5c	Describe roles of funder(s), sponsor(s), and / or institution(s), if any, in developing the protocol	12
**Introduction**			

For peer review only

Rationale	#6	Describe the rationale for the review in the context of what is already known	3-5
Objectives	#7	Provide an explicit statement of the question(s) the review will address with reference to participants, interventions, comparators, and outcomes (PICO)	5
**Methods**			
Eligibility criteria	#8	Specify the study characteristics (such as PICO, study design, setting, time frame) and report characteristics (such as years considered, language, publication status) to be used as criteria for eligibility for the review	5-7
Information sources	#9	Describe all intended information sources (such as electronic databases, contact with study authors, trial registers or other grey literature sources) with planned dates of coverage	7
Search strategy	#10	Present draft of search strategy to be used for at least one electronic database, including planned limits, such that it could be repeated	8
Study records - data management	#11a	Describe the mechanism(s) that will be used to manage records and data throughout the review	8
Study records - selection process	#11b	State the process that will be used for selecting studies (such as two independent reviewers) through each phase of the review (that is, screening, eligibility and inclusion in meta-analysis)	8

For peer review only

Study records - data collection process	#11c	Describe planned method of extracting data from reports (such as piloting forms, done independently, in duplicate), any processes for obtaining and confirming data from investigators	8
Data items	#12	List and define all variables for which data will be sought (such as PICO items, funding sources), any pre-planned data assumptions and simplifications	8,9
Outcomes and prioritization	#13	List and define all outcomes for which data will be sought, including prioritization of main and additional outcomes, with rationale	6,7
Risk of bias in individual studies	#14	Describe anticipated methods for assessing risk of bias of individual studies, including whether this will be done at the outcome or study level, or both; state how this information will be used in data synthesis	9
Data synthesis	#15a	Describe criteria under which study data will be quantitatively synthesised	9
Data synthesis	#15b	If data are appropriate for quantitative synthesis, describe planned summary measures, methods of handling data and methods of combining data from studies, including any planned exploration of consistency (such as I2, Kendall's τ)	9,10
Data synthesis	#15c	Describe any proposed additional analyses (such as sensitivity or subgroup analyses, meta-regression)	9,10

For peer review only

Data synthesis	#15d	If quantitative synthesis is not appropriate, describe the type of summary planned	9,10
Meta-bias(es)	#16	Specify any planned assessment of meta-bias(es) (such as publication bias across studies, selective reporting within studies)	11
Confidence in cumulative evidence	#17	Describe how the strength of the body of evidence will be assessed (such as GRADE)	9

The PRISMA-P checklist is distributed under the terms of the Creative Commons Attribution License CC-BY 4.0. This checklist was completed on 20. November 2019 using https://www.goodreports.org/, a tool made by the EQUATOR Network in collaboration with Penelope.ai

For peer review only

BMJ Open

HEALTH SERVICE USE AMONG ADULTS WITH CEREBRAL PALSY: A MIXED METHODS SYSTEMATIC REVIEW PROTOCOL.

Journal:	*BMJ Open*
Manuscript ID	bmjopen-2019-035892.R1
Article Type:	Protocol
Date Submitted by the Author:	18-Mar-2020
Complete List of Authors:	Manikandan, Manjula; Royal College of Surgeons in Ireland, Department of Public Health and Epidemiology<br>Walsh, Aisling; Royal College of Surgeons in Ireland, Department of Public Health and Epidemiology<br>Kerr, C ; Queen's University Belfast, School of Nursing and Midwifery<br>Walsh, Michael; Health Service Executive, Office of the Chief Clinical Officer<br>Ryan, Jennifer; Brunel University London, College of Health and Life Sciences; Royal College of Surgeons in Ireland, Department of Public Health and Epidemiology
**Primary Subject Heading**:	Health services research
Secondary Subject Heading:	Neurology
Keywords:	Adult neurology < NEUROLOGY, REHABILITATION MEDICINE, HEALTH SERVICES ADMINISTRATION & MANAGEMENT

SCHOLARONE™
Manuscripts

I, the Submitting Author has the right to grant and does grant on behalf of all authors of the Work (as defined in the below author licence), an exclusive licence and/or a non-exclusive licence for contributions from authors who are: i) UK Crown employees; ii) where BMJ has agreed a CC-BY licence shall apply, and/or iii) in accordance with the terms applicable for US Federal Government officers or employees acting as part of their official duties; on a worldwide, perpetual, irrevocable, royalty-free basis to BMJ Publishing Group Ltd ("BMJ") its licensees and where the relevant Journal is co-owned by BMJ to the co-owners of the Journal, to publish the Work in this journal and any other BMJ products and to exploit all rights, as set out in our licence.

The Submitting Author accepts and understands that any supply made under these terms is made by BMJ to the Submitting Author unless you are acting as an employee on behalf of your employer or a postgraduate student of an affiliated institution which is paying any applicable article publishing charge ("APC") for Open Access articles. Where the Submitting Author wishes to make the Work available on an Open Access basis (and intends to pay the relevant APC), the terms of reuse of such Open Access shall be governed by a Creative Commons licence – details of these licences and which Creative Commons licence will apply to this Work are set out in our licence referred to above.

Other than as permitted in any relevant BMJ Author's Self Archiving Policies, I confirm this Work has not been accepted for publication elsewhere, is not being considered for publication elsewhere and does not duplicate material already published. I confirm all authors consent to publication of this Work and authorise the granting of this licence.

For peer review only

HEALTH SERVICE USE AMONG ADULTS WITH CEREBRAL PALSY: A MIXED METHODS SYSTEMATIC REVIEW PROTOCOL.

Manjula Manikandan[1], Aisling Walsh[1], Claire Kerr[2], Michael Walsh[4], Jennifer Ryan[1, 3]

[1]Department of Public Health and Epidemiology, Royal College of Surgeons in Ireland, Ireland

[2]School of Nursing and Midwifery, Queen's University Belfast, United Kingdom

[3]College of Health and Life Sciences, Brunel University London, United Kingdom

[4]Office of the Chief Clinical Officer, Health Service Executive, Ireland

Email: manjulamanikandan@rcsi.ie, aislingwalsh@rcsi.ie, c.kerr@qub.ac.uk, michael.walsh14@hse.ie, jenniferryan@rcsi.ie

Corresponding author: Jennifer Ryan and Manjula Manikandan; RCSI Department of Public Health and Epidemiology, Royal College of Surgeons in Ireland, Lower Mercer Street, Dublin 2, Ireland.

Telephone number of the corresponding author: +353 1402 2413; +353 1402 8507

Keywords: adults; cerebral palsy; health service use; experiences; protocol; systematic review

Word count: 2859 words (excluding references)

Abstract word count: 317 words

For peer review only - http://bmjopen.bmj.com/site/about/guidelines.xhtml

ABSTRACT

Introduction: Cerebral palsy (CP) is a childhood neurodisability that primarily results in motor impairments and activity limitations, but is often associated with epilepsy and disturbances of sensation, perception, cognition, behaviour and speech. Most children with CP survive well into adulthood. Adults with CP experience increased risk of age related chronic conditions such as arthritis, stroke, cardio respiratory and mental health conditions in addition to the ongoing disabilities experienced from childhood. Therefore, adults with CP often require extensive health services. However, health service use among adults with CP has not been well documented. This mixed method review aims to identify, appraise and synthesise quantitative and qualitative literature examining health service use among adults with CP.

Methods and analysis: The mixed method systematic review will be conducted in accordance with the Joanna Briggs Institute (JBI) methodology. A systematic search of Medline (Ovid), CINAHL, EMBASE, PsychINFO and Cochrane library from inception to March 2020 will be conducted. Quantitative observational studies, qualitative studies, and mixed method studies examining health service use among adults with CP (≥ 18 years) will be included. Outcomes of interest are the frequency and proportion of adults using health services and the experiences of health service use from the perspectives of adults with CP, caregivers and health service providers. . Two reviewers will independently screen titles, abstracts and full texts, extract data, and assess the quality of included studies using instruments from the JBI. Where possible a pooled analysis and aggregation of findings will be performed for quantitative and qualitative data, respectively. The quantitative and qualitative findings will be integrated using a triangulation approach at the synthesis stage. A narrative synthesis will be carried out where this is not possible.

Ethics and dissemination: Ethics approval is not required for this review. The findings will be disseminated through a peer-reviewed journal and conferences.

For peer review only - http://bmjopen.bmj.com/site/about/guidelines.xhtml

Systematic review registration number: The protocol is under review at PROSPERO [receipt number: 155380].

STRENGTHS & LIMITATIONS OF THIS STUDY

- This mixed method systematic review will include quantitative, qualitative and mixed methods studies in order to provide a comprehensive overview of the current literature relating to health services for adults with cerebral palsy.
- This review process is strengthened by utilisation of the widely used Andersen-Newman behavioural model of health service utilisation as an overarching framework to guide data extraction and analysis.
- Only studies that are published in English will be included in the review.
- Heterogeneity of eligible studies may limit the possibility of meta-analysis.

BACKGROUND

Cerebral palsy (CP) is a "group of permanent disorders of movement and posture, causing activity limitation, that are linked to non-progressive disturbances of the developing fetal or infant brain" [1].The disturbance to the brain can occur before, during or up to approximately 2 years after birth from traumatic or non-traumatic brain injury [2 3] . With recent medical advances, CP can be diagnosed accurately before the age of 6 months [4]. People with CP present with different types of motor abnormality, namely spasticity, dyskinesia and ataxia [1 5]. In addition to movement disorders, people with CP may experience epilepsy or impairments of cognition, speech, hearing and vision [1 5].

Traditionally, CP was considered a paediatric condition. The incidence rate of CP remains steady between 2.0-2.5 cases per 1000 live births, however recently the birth prevalence has decreased in Europe and Australia [6-9]. Many people with CP survive well into adulthood with a recent study reporting more than 80% of people with CP have a life expectancy, beyond 58 years [10]. People with CP continue to present

For peer review only - http://bmjopen.bmj.com/site/about/guidelines.xhtml

with ongoing health issues from childhood, in addition to the issues related to ageing. For instance, adults with CP experience increased risk of arthritis, stroke, heart failure, ischaemic heart disease, chronic respiratory disease and mental health disorders compared to adults without CP [11-14]. Studies have also reported increased fatigue, incidence of falls, pain and a decline in mobility, as people with CP move into adulthood [15-17]. Therefore, adults with CP may require extensive health services.

The National Institute for Health and Care Excellence (NICE) guideline for cerebral palsy in adults has recommended continuity of care for adults with CP, tailored to their needs [18]. This continuity of care is likely to avoid emergency hospital admissions[18]. However, studies of adolescents with CP have reported challenging experiences in navigating services during transition from child to adult services [19 20]. In addition, adolescents with CP reported anxiety about transfer from child to adult services as they perceived that services in adulthood were poorly co-ordinated and managed [19-21]. As the evidence describing the long-term health issues experienced by adults with CP has expanded in recent years, there is a growing need for research examining health service use among adults with CP.

'Health services' are defined as those "services that are first and foremost undertaken to have direct effect on people's health. These extend from health promotion and disease prevention, through curative services, to long term care and rehabilitation"[22]. Use of health services may be associated with various factors relating to the individual and their environment [23 24]. The previous Andersen-Newman behavioural models of health service utilisation (ANM) proposed family and individual as the unit of analysis for health service use [24]. The revised Andersen-Newman behavioural model of health service utilisation (ANM) provides a framework for considering health service use [23]. It describes environmental factors, population factors and outcomes that can be associated with health service use behaviour [23]. Environmental factors

For peer review only - http://bmjopen.bmj.com/site/about/guidelines.xhtml

include those relating to the health care system (e.g., national policies, resources and organisations involved) and external environment (e.g., physical, political and economic factors) [23]. Population factors include demographics (e.g. age, gender, ethnicity, location and condition-specific characteristics such as functional mobility and type of motor abnormality) [23]. Outcomes include perceived and evaluated health status and customer satisfaction [23].Over the years, ANM has been adapted, critiqued and reviewed extensively [25-29]. However, the revised ANM (1995) includes environment as a key driver of the model impacting health service use behaviour [23]. It also emphasizes the relationship between factors through feedback loops, which demonstrates the complexity involved in health service use behaviour [23] .

A preliminary search of PROSPERO, MEDLINE (Ovid), JBI database and the Cochrane Database of Systematic Reviews indicated that no systematic reviews have been conducted examining health service use among adults with CP. Previous reviews have focused on the epidemiology of CP in adults, quality of life, pain and effectiveness of interventions used by adults with CP[16 30-32]. There is a need for a theory informed review with broader perspectives on health service use among adults with CP, rather than focusing on the effectiveness of any specific intervention. Such a review would potentially inform health service used by adults with CP, direct research efforts, inform service delivery and identify areas of challenges faced by adults with CP. Therefore, the aim of this mixed method systematic review is to identify, appraise and synthesise the available quantitative and qualitative literature examining health service use among adults with CP using the ANM as a framework.

OBJECTIVES

The objectives are:

1. To determine the proportion and frequency of health service use by adults with CP.
2. To examine associations between environmental factors, population factors, outcomes and health service use.

For peer review only - http://bmjopen.bmj.com/site/about/guidelines.xhtml

3. To explore the experiences and perceptions of health service use for adults with CP, from the perspective of adults with CP, caregivers and health service providers.

METHODS

This mixed method systematic review will be conducted in accordance with the Joanna Briggs Institute methodology guidelines [33]. The protocol was submitted for registration on the PROSPERO database of systematic reviews [receipt number: 155380]. This review is written according to the PRISMA-P checklist [34]. The review will be reported using MOOSE and ENTREQ reporting guidelines [35 36].

Study eligibility

Study design

The review will include quantitative, qualitative and mixed method studies. Quantitative studies will include observational studies (e.g., cross-sectional, case-control and cohort studies). Qualitative studies will include studies of recognised qualitative approaches (e.g., interviews, focus groups, observation, thematic analysis, content analysis, narrative analysis and framework analysis) [37]. Mixed method studies will only be considered if quantitative or qualitative components can be clearly extracted.

Studies published in English language will be included. However, there will be no restriction on the geographic location of studies or date of publication

Participants

Studies of adults with CP (≥ 18 years) with all types of motor disorders and all levels of functional mobility will be included for both quantitative and qualitative studies. Functional mobility for people with CP is widely classified using the Gross Motor Function Classification System (GMFCS) [38]. For qualitative studies, articles including caregivers (also includes family members) and health service providers will also be included. For studies that include children and young adults (for example, those aged 15-25 years), data

For peer review only

from people ≥18 years only will be included, if possible. If it is not possible to extract data for adults only, we will include data if the mean age of the sample is ≥18 years. Where studies include data on adults with CP as part of a larger sample of people with disabilities, we will extract data on adults with CP only, if possible.

Outcome

Health services for this review will include services that have direct impact on the health of adults with CP [22 39-42]. This can include hospital admissions, emergency department visits, outpatient visits to medical and allied health professionals (e.g. physicians, pharmacist, physiotherapists, occupational therapists, podiatrists, psychologists, orthotists, speech and language therapists). In addition, diagnostic or assistive device services (e.g. radiologists, communication aids, mobility aids and wheelchair services) and support services (e.g. supported living, personal assistance, residential, respite and domiciliary services) will also be included [22 39-42].

For the quantitative component of this review, the proportion of adults using health services and the frequency of health service use will be the main outcomes. Where possible, we will report each type of health services used by adults with CP.

For the qualitative component, the main outcomes are experience and perceptions of using health services among adults with CP. We will also explore experiences and perceptions of health services for adults with CP from the perspectives of caregivers, and health service providers.

Exclusion criteria

We will exclude reviews, dissertations, editorials, commentaries and conference abstracts. Studies of children and young people with CP (<18 years) will be excluded. Studies examining services that have an indirect effect on people's health only such as education, employment, housing and transport will be

For peer review only - http://bmjopen.bmj.com/site/about/guidelines.xhtml

excluded [22]. Finally, randomised controlled studies on intervention effectiveness, pragmatic, open-label trials, case series and case reports will be excluded from this review.

Search Strategy

The electronic databases Medline (Ovid), CINAHL, EMBASE, PsychINFO and Cochrane library will be systematically searched from inception of each database to March 2020. We will conduct a pilot search in Medline (Ovid) to inform the search strategy. A librarian will support development of the search strategy. Reference lists of included studies will be screened.

Search terms

The search strategy will consist of fully exploded subject headings and free text words relating to adults with CP and health services. The search strategy developed from Medline (Ovid) will be tailored to each database. No filters on study design or settings will be applied at this stage. Search terms will be combined using Boolean operators. A sample search strategy for Medline (Ovid) is provided in Appendix 1.

Data management

A search log will be maintained to record the databases, keywords used and results of each search. The titles and abstracts of papers identified from the systematic search will be imported into reference management software (Endnote X8.2) and duplicates will be removed.

Study selection

Two reviewers will screen titles and abstracts independently using Rayyan software [43]. Where studies meet the inclusion criteria or it is unclear if they are eligible, full-texts will be retrieved for further assessment. The two reviewers will screen full text articles for inclusion independently. Conflicts will be resolved by discussion or third reviewer involvement. The reasons for exclusion of full text studies will be

For peer review only - http://bmjopen.bmj.com/site/about/guidelines.xhtml

recorded and the summary of the search results will be presented in a Preferred Reporting Items for Systematic Reviews and Meta-analyses (PRISMA) flow diagram [44].

Data extraction

Two reviewers will extract data independently using the JBI data extraction tool for quantitative and qualitative studies accordingly. Any disagreements will be resolved initially through discussion and if required, consultation with a third reviewer. The study authors will be contacted to retrieve missing data necessary for meta-analysis or meta-aggregation.

Data items

For quantitative studies and quantitative components of mixed method studies, extracted data will include study details, study design, country, funding sources, declaration of interests, participant characteristics, exposure and outcome of interest [45]. This will include proportion of people using each health service, frequency of use of each health service, and variables relating to the environmental factors, population factors and outcomes according to the ANM. For qualitative studies and the qualitative components of mixed method studies, the extracted data will include study design, setting, country, methodology, funding sources, declaration of interests, participants, data analysis and themes related to experiences [46]. The qualitative data, such as quotes or themes of experiences and perceptions will be extracted from the results sections of the included studies.

Quality assessment

Two reviewers will independently assess the methodological quality of included studies using standardised critical appraisal instruments from the Joanna Briggs Institute[33]. For quantitative studies the

For peer review only - http://bmjopen.bmj.com/site/about/guidelines.xhtml

appropriate JBI- critical appraisal checklist for cohort, case-control and analytical cross-sectional studies will be used [45]. For qualitative studies the JBI- qualitative appraisal instrument will be used [46]. Quality appraisal will be undertaken at study level and studies will not be excluded for data extraction or synthesis based on the methodological quality assessment, as this review seeks to capture all experiences of health service use by people with CP, and so necessitates inclusion of studies of lower methodological quality. Disagreements between the reviewers will be discussed and discrepancies resolved by a third reviewer. The results of the quality appraisal will be reported in a table and narrative form.

GRADE will be used to interpret the quantitative review findings and demonstrate the overall quality of the data [47]. GRADE-CERQual will be used to interpret the qualitative review findings in the context of methodological limitations, relevance, coherence and adequacy of data [48]. According to the JBI guidelines for mixed methods review, there are complexities involved in assessing confidence in findings from combined quantitative and qualitative evidence [33]. Therefore, GRADE approach will only be used for separate qualitative and quantitative evidence analysis, but not for assessing the integrated findings.

Data synthesis

*Quantitative studies*The pooled proportion of people using each type of health services and pooled mean visits to each type of health service will be estimated using a random effects model. We will standardise frequency to a specific time frame. Separate meta-analyses will be conducted for each service. We will use random-effects model due to the likely heterogeneity of the population and outcome variable [49]. We will assess statistical heterogeneity using the I^2 statistic and a Chi^2 test [50]. If studies compare health service use between people with and without CP, we will calculate an odds ratio for dichotomous data and a standardized mean difference for continuous data. Where data cannot be pooled, a narrative synthesis of the quantitative findings will be conducted.

For peer review only - http://bmjopen.bmj.com/site/about/guidelines.xhtml

Qualitative studies

Qualitative experiences from different perspectives will be pooled using a meta-aggregation approach [51]. This involves identifying the findings, categorising the findings based on similarity in meaning and grouping the categories into synthesised findings [51]. Where textual-pooling is not possible, the findings will be presented in narrative form.

Integration

The findings from quantitative and qualitative synthesis will be integrated using a convergent segregated approach, to preserve the integrity of quantitative and qualitative findings [33]. The results from quantitative and qualitative components will be triangulated and reported using the ANM as a framework. Triangulation will involve comparing or organising quantitative and qualitative findings into a line of argument via tabulation, groupings and visual representation to produce an overall configured analysis [52]. A narrative form of discussion will be considered, where this is not possible.

Patient and Public Involvement (PPI)

The patient and public were not involved in developing and designing of this review protocol. However, PPI panel members of adults with CP and professionals will be involved in interpretation and dissemination of the review findings.

ETHICS AND DISSEMINATION

Ethical approval is not required for this review as both quantitative and qualitative data used for analysis will be extracted from published studies. The review will be published in a peer-reviewed journal and presented at conferences for researchers, health professionals and people with CP. Findings will be disseminated to healthcare providers and shared with charity and voluntary agencies that support people with CP.

For peer review only - http://bmjopen.bmj.com/site/about/guidelines.xhtml

DISCUSSION

This mixed methods review will provide greater insights of the available literature on the health services used by adults with CP and related experiences of health services from the perspectives of adults with CP, their caregivers and health service providers. The majority of people with CP will survive to at least 60 years of age [10]. Many adults with CP experience secondary conditions with age that require intervention and support from clinicians working across multiple health and social care disciplines[12]. Previous research has demonstrated that young people with CP experience challenges in accessing health services, as they move from child to adult health services [19 21 53-55]. Therefore, a review of health service use among adults with CP is required in order to direct research efforts and inform service provision.

A theory-informed review using the revised ANM will guide examination of factors associated with health service use among adults with CP[23]. This model describes factors by environment, population and outcomes influencing health service use behaviour with feedback loops showing the complexities involved[23]. However, the ANM provides only a cross-sectional view on health service use and does not include change or life course as a factor [29]. This incomplete view of a phenomenon is common among most theoretical frameworks used in the literature [56]. The life course perspective provides a complete understanding of past versus present service use and the changes seen over time[29]. However, where possible a narrative synthesis will be done for life course or change over time in service use.

A limitation of this review is the exclusion of studies that are not peer-reviewed or published in languages other than English, potentially resulting in less generalisable findings from other languages. Finally, it may not be possible to conduct a meta-analysis due to a lack of / and/or heterogeneity of data.

For peer review only - http://bmjopen.bmj.com/site/about/guidelines.xhtml

AUTHOR CONTRIBUTIONS

All authors have agreed on the final version and meet at least two of the following criteria (recommended by ICMJE www.icmje.org/recommendations):

1. Substantial contributions to the conception or design of the work; or the acquisition, analysis, or interpretation of data for the work **(MM, AW, CK, MW and JR)**
2. Drafting the work or revising it critically for important intellectual content (**MM, JR)**
3. Final approval of the version to be published **(MM, AW, CK, MW and JR)**
4. Agreement to be accountable for all aspects of the work in ensuring that questions related to the accuracy or integrity of any part of the work are appropriately investigated and resolved **(MM and JR).**

ACKNOWLEDGEMENTS

The authors acknowledge the support from the RCSI librarian (Ms Grainne McCabe) for supporting the development of search terms with the reviewers.

COMPETING INTERESTS

The authors disclose no competing interests.

FUNDING

This work was conducted as part of the SPHeRE Programme under Grant No. SPHeRE/2013/1. This review is funded by the Royal College of Surgeons in Ireland (RCSI) through the StAR programme. The funders had no role in developing the protocol.

For peer review only

REFERENCES

1. Colver A, Fairhurst C, Pharoah PO. Cerebral palsy. *Lancet (London, England)* 2014;383(9924):1240-49. doi: 10.1016/s0140-6736(13)61835-8 [published Online First: 2013/11/26]

2. McIntyre S, Morgan C, Walker K, et al. Cerebral Palsy—Don't Delay. *Developmental Disabilities Research Reviews* 2011;17(2):114-29. doi: 10.1002/ddrr.1106

3. Granild-Jensen JB, Rackauskaite G, Flachs EM, et al. Predictors for early diagnosis of cerebral palsy from national registry data. *Developmental Medicine & Child Neurology* 2015;57(10):931-35. doi: 10.1111/dmcn.12760

4. Novak I, Morgan C, Adde L, et al. Early, Accurate Diagnosis and Early Intervention in Cerebral Palsy: Advances in Diagnosis and Treatment. *JAMA Pediatrics* 2017;171(9):897-907. doi: 10.1001/jamapediatrics.2017.1689 [published Online First: 2017/07/18]

5. Novak I, Hines M, Goldsmith S, et al. Clinical prognostic messages from a systematic review on cerebral palsy. *Pediatrics* 2012;130(5):e1285-312. doi: 10.1542/peds.2012-0924 [published Online First: 2012/10/10]

6. Johnson A. Prevalence and characteristics of children with cerebral palsy in Europe. *Developmental Medicine & Child Neurology* 2002;44(9):633-40. doi: 10.1017/S0012162201002675 [published Online First: 2002/09/16]

7. Oskoui M, Coutinho F, Dykeman J, et al. An update on the prevalence of cerebral palsy: a systematic review and meta-analysis. *Developmental Medicine & Child Neurology* 2013;55(6):509-19. doi: 10.1111/dmcn.12080 [published Online First: 2013/01/26]

8. Galea C, Mcintyre S, Smithers-Sheedy H, et al. Cerebral palsy trends in Australia (1995–2009): a population-based observational study. *Developmental Medicine & Child Neurology* 2019;61(2):186-93. doi: 10.1111/dmcn.14011

9. Sellier E, Platt MJ, Andersen GL, et al. Decreasing prevalence in cerebral palsy: a multi-site European population-based study, 1980 to 2003. *Developmental Medicine & Child Neurology* 2016;58(1):85-92. doi: 10.1111/dmcn.12865

For peer review only - http://bmjopen.bmj.com/site/about/guidelines.xhtml

10. Blair E, Langdon K, McIntyre S, et al. Survival and mortality in cerebral palsy: observations to the sixth decade from a data linkage study of a total population register and National Death Index. *BMC Neurology* 2019;19(1):111.

11. Peterson MD, Ryan JM, Hurvitz EA, et al. Chronic Conditions in Adults With Cerebral Palsy. *JAMA* 2015;314(21):2303-5. doi: 10.1001/jama.2015.11025 [published Online First: 2015/12/02]

12. Ryan JM, Peterson MD, Matthews A, et al. Noncommunicable disease among adults with cerebral palsy: A matched cohort study. *Neurology* 2019;93(14):e1385-e96.

13. Smith KJ, Peterson MD, O'Connell NE, et al. Risk of depression and anxiety in adults with cerebral palsy. *JAMA Neurology* 2019;76(3):294-300.

14. O'Connell NE, Smith KJ, Peterson MD, et al. Incidence of osteoarthritis, osteoporosis and inflammatory musculoskeletal diseases in adults with cerebral palsy: A population-based cohort study. *Bone* 2019;125:30-35.

15. Bottos M, Feliciangeli A, Sciuto L, et al. Functional status of adults with cerebral palsy and implications for treatment of children. *Developmental Medicine & Child Neurology* 2001;43(8):516-28.

16. van Gorp M, Hilberink SR, Noten S, et al. The epidemiology of cerebral palsy in adulthood: A systematic review and meta-analysis of the most frequently studied outcomes. *Archives of Physical Medicine and Rehabilitation* 2020

17. Ryan JM, Cameron MH, Liverani S, et al. Incidence of falls among adults with cerebral palsy: a cohort study using primary care data. *Developmental Medicine & Child Neurology* 2020;62(4):477-82. doi: 10.1111/dmcn.14444 [published Online First: 2019/12/28]

18. National Institute for Health and Care Excellence (NICE). Cerebral Palsy in adults London 2019 [Available from: https://www.nice.org.uk/guidance/ng119/chapter/Rationale-and-impact.

For peer review only - http://bmjopen.bmj.com/site/about/guidelines.xhtml

19. Hislop J, Mason H, Parr JR, et al. Views of Young People With Chronic Conditions on Transition From Pediatric to Adult Health Services. *Journal of Adolescent Health* 2016;59(3):345-53. doi: https://doi.org/10.1016/j.jadohealth.2016.04.004

20. Wright AE, Robb J, Shearer MC. Transition from paediatric to adult health services in Scotland for young people with cerebral palsy. *Journal of child health care : for professionals working with children in the hospital and community* 2016;20(2):205-13. doi: 10.1177/1367493514564632 [published Online First: 2015/01/17]

21. Colver A, Pearse R, Watson RM, et al. How well do services for young people with long term conditions deliver features proposed to improve transition? *BMC Health Service Research* 2018;18(1):337. doi: 10.1186/s12913-018-3168-9 [published Online First: 2018/05/10]

22. Black N, Gruen R. Understanding health services: McGraw-Hill Education (UK) 2005.

23. Andersen RM. Revisiting the behavioral model and access to medical care: does it matter? *Journal of Health and Social Behavior* 1995:1-10.

24. Andersen R, Newman JF. Societal and individual determinants of medical care utilization in the United States. *The Milbank Memorial Fund Quarterly Health and Society* 1973:95-124.

25. Goldsmith LJ. Access to health care for disadvantaged individuals: A qualitative inquiry. 2007

26. Babitsch B, Gohl D, von Lengerke T. Re-revisiting Andersen's Behavioral Model of Health Services Use: a systematic review of studies from 1998-2011. *Psychosoc Med* 2012;9:Doc11-Doc11. doi: 10.3205/psm000089 [published Online First: 2012/10/25]

27. Guilcher SJ, Craven BC, McColl MA, et al. Application of the Andersen's health care utilization framework to secondary complications of spinal cord injury: a scoping review. *Disability and Rehabilitation* 2012;34(7):531-41.

28. Phillips KA, Morrison KR, Andersen R, et al. Understanding the context of healthcare utilization: assessing environmental and provider-related variables in the behavioral model of utilization. *Health Services Research* 1998;33(3 Pt 1):571.

For peer review only

For peer review only - http://bmjopen.bmj.com/site/about/guidelines.xhtml

29. O'Donovan M-A, McCallion P, McCarron M, et al. A narrative synthesis scoping review of life course domains within health service utilisation frameworks. *HRB Open Research* 2019;2(6):6.

30. Alves-Nogueira A, Silva N, McConachie H, et al. A systematic review on quality of life assessment in adults with cerebral palsy: Challenging issues and a call for research. *Research in Developmental Disabilities* 2020;96:103514.

31. van der Slot WM, Benner JL, Brunton L, et al. Pain in adults with cerebral palsy: a systematic review and meta-analysis of individual participant data. *Annals of Physical and Rehabilitation Medicine* 2020

32. Adams CT, Lakra A. Clinical and Functional Outcomes of Total Hip Arthroplasty in Patients with Cerebral Palsy: A Systematic Review. *Journal of Orthopaedics* 2020

33. Lizarondo L SC, Carrier J, Godfrey C, Rieger K, Salmond S, Apostolo J, Kirkpatrick P, Loveday, Hadders-Algra, M. Chapter 8: Mixed methods systematic reviews. In: Aromataris E, Munn Z (Editors). Joanna Briggs Institute Reviewer's Manual. The Joanna Briggs Institute 2017 [Available from: https://reviewersmanual.joannabriggs.org/.

34. Moher D, Shamseer L, Clarke M, et al. Preferred reporting items for systematic review and meta-analysis protocols (PRISMA-P) 2015 statement. *Systematic Reviews* 2015;4(1):1.

35. Tong A, Flemming K, McInnes E, et al. Enhancing transparency in reporting the synthesis of qualitative research: ENTREQ. *BMC Medical Research Methodology* 2012;12:181. doi: 10.1186/1471-2288-12-181 [published Online First: 2012/11/29]

36. Stroup DF, Berlin JA, Morton SC, et al. Meta-analysis of observational studies in epidemiology: a proposal for reporting. Meta-analysis Of Observational Studies in Epidemiology (MOOSE) group. *JAMA* 2000;283(15):2008-12. [published Online First: 2000/05/02]

37. Creswell JW, Poth CN. Qualitative inquiry and research design: Choosing among five approaches. 4th ed: Sage publications 2017.

38. Palisano R, Rosenbaum P, Walter S, et al. Development and reliability of a system to classify gross motor function in children with cerebral palsy. *Developmental Medicine and Child*

For peer review only - http://bmjopen.bmj.com/site/about/guidelines.xhtml

Neurology 1997;39(4):214-23. doi: 10.1111/j.1469-8749.1997.tb07414.x [published Online First: 1997/04/01]

39. Cheng SM, Catallo C. Case definition for health and social care services integrated initiatives. *Journal of Integrated Care* 2019

40. Leutz WN. Five laws for integrating medical and social services: lessons from the United States and the United Kingdom. *The Milbank Quarterly* 1999;77(1):77-110.

41. Leichsenring K. Developing integrated health and social care services for older persons in Europe. *International Journal of Integrated Care* 2004;4:e10-e10. doi: 10.5334/ijic.107

42. Dickens J. The definition of social work in the United Kingdom, 2000–2010. *International Journal of Social Welfare* 2012;21(1):34-43. doi: 10.1111/j.1468-2397.2011.00782.x

43. Ouzzani M, Hammady H, Fedorowicz Z, et al. Rayyan-a web and mobile app for systematic reviews. *Systematic Reviews* 2016;5(1):210-10. doi: 10.1186/s13643-016-0384-4

44. Moher D, Liberati A, Tetzlaff J, et al. Preferred reporting items for systematic reviews and meta-analyses: the PRISMA statement. *Annals of Internal Medicine* 2009;151(4):264-69.

45. Moola S MZ, Tufanaru C, Aromataris E, Sears K, Sfetcu R, Currie M, Lisy K, Qureshi R, Mattis P, Mu P. Chapter 7: Systematic reviews of etiology and risk. In: Aromataris E, Munn Z (Editors). Joanna Briggs Institute Reviewer's Manual. The Joanna Briggs Institute. 2017 [Available from: https://reviewersmanual.joannabriggs.org/

46. Lockwood C PK, Munn Z, Rittenmeyer L, Salmond S, Bjerrum M, Loveday H, Carrier J, Stannard D. Chapter 2: Systematic reviews of qualitative evidence. In: Aromataris E, Munn Z (Editors). Joanna Briggs Institute Reviewer's Manual. The Joanna Briggs Institute. 2017 [Available from: https://reviewersmanual.joannabriggs.org/.

47. GRADE. Grading of Recommendations Assessment, Development and Evaluation GRADE working group [Available from: https://www.gradeworkinggroup.org/ accessed 03/03/2020.

For peer review only - http://bmjopen.bmj.com/site/about/guidelines.xhtml

48. Lewin S, Glenton C, Munthe-Kaas H, et al. Using qualitative evidence in decision making for health and social interventions: an approach to assess confidence in findings from qualitative evidence syntheses (GRADE-CERQual). *PLOS Medicine* 2015;12(10)

49. Borenstein M, Hedges LV, Higgins JPT, et al. A basic introduction to fixed-effect and random-effects models for meta-analysis. *Research Synthesis Methods* 2010;1(2):97-111. doi: 10.1002/jrsm.12

50. Munn Z, Tufanaru C, Aromataris E. JBI's systematic reviews: data extraction and synthesis. *AJN The American Journal of Nursing* 2014;114(7):49-54.

51. Pearson A. Balancing the evidence: incorporating the synthesis of qualitative data into systematic reviews. *JBI reports* 2004;2(2):45-64.

52. Noyes J, Booth A, Moore G, et al. Synthesising quantitative and qualitative evidence to inform guidelines on complex interventions: clarifying the purposes, designs and outlining some methods. *BMJ Global Health* 2019;4(Suppl 1):e000893.

53. Colver A, McConachie H, Le Couteur A, et al. A longitudinal, observational study of the features of transitional healthcare associated with better outcomes for young people with long-term conditions. *BMC Medicine* 2018;16(1):111.

54. Bagatell N, Chan D, Rauch KK, et al. “Thrust into adulthood”: Transition experiences of young adults with cerebral palsy. *Disability and Health Journal* 2017;10(1):80-86. doi: https://doi.org/10.1016/j.dhjo.2016.09.008

55. Kolehmainen N, McCafferty S, Maniatopoulos G, et al. What constitutes successful commissioning of transition from children’s to adults’ services for young people with long-term conditions and what are the challenges? An interview study. *BMJ Paediatrics Open* 2017;1(1) doi: 10.1136/bmjpo-2017-000085

56. Shannon-Baker P. Making paradigms meaningful in mixed methods research. *Journal of Mixed Methods Research* 2016;10(4):319-34.

For peer review only - http://bmjopen.bmj.com/site/about/guidelines.xhtml

Appendix 1

Medline (Ovid)

Ovid MEDLINE(R) and Epub Ahead of Print, In-Process & Other Non-Indexed Citations, Daily and Versions(R) 1946 to present

1. exp Cerebral Palsy/
2. cerebral pals$.tw.
3. ((Hemiplegi$ or diplegi$ or quadriplegi$ or unilateral$) adj5 spastic$).tw.
4. ((Hemiplegi$ or diplegi$ or quadriplegi$ or unilateral$) adj3 ataxi$).tw.
5. Little$ disease.tw.
6. 1 or 2 or 3 or 4 or 5

7. exp Health Services/
8. exp Health Facilities/
9. exp Comprehensive Health Care/
10. exp "Delivery of Health Care"/
11. exp "analytical, diagnostic and therapeutic techniques and equipment (non mesh)"/
12. exp residential treatment/
13. emergenc$.tw.
14. therap$.tw.
15. diagnos$.tw.
16. assistive device$.tw.

For peer review only

17. health service$.tw.

18. health care$.tw.

19. hospital$.tw.

20. rehabilitation$.tw.

21. general practice$.tw.

22. family practice$.tw.

23. primary care$.tw.

24. community$.tw.

25. respite$.tw.

26. residential care$.tw

27. 7 or 8 or 9 or 10 or 11 or 12 or 13 or 14 or 15 or 16 or 17 or 18 or 19 or 20 or 21 or 22 or 23 or 24 or 25 or 26

28. exp Adult/

29. adult$.tw.

30. elder$.tw.

31. older$.tw.

32. young adult$.tw.

33. 28 or 29 or 30 or 31 or 32

34. 6 AND 27 AND 33

For peer review only

Reporting checklist for protocol of a systematic review.

Based on the PRISMA-P guidelines.

Instructions to authors

Complete this checklist by entering the page numbers from your manuscript where readers will find each of the items listed below.

Your article may not currently address all the items on the checklist. Please modify your text to include the missing information. If you are certain that an item does not apply, please write "n/a" and provide a short explanation.

Upload your completed checklist as an extra file when you submit to a journal.

In your methods section, say that you used the PRISMA-Preporting guidelines, and cite them as:

Moher D, Shamseer L, Clarke M, Ghersi D, Liberati A, Petticrew M, Shekelle P, Stewart LA. Preferred Reporting Items for Systematic Review and Meta-Analysis Protocols (PRISMA-P) 2015 statement. Syst Rev. 2015;4(1):1.

		Reporting Item	Page Number
**Title**			
Identification	#1a	Identify the report as a protocol of a systematic review	1
Update	#1b	If the protocol is for an update of a previous systematic review, identify as such	n/a

For peer review only

Registration

	#2	If registered, provide the name of the registry (such as PROSPERO) and registration number	3

Authors

Contact	#3a	Provide name, institutional affiliation, e-mail address of all protocol authors; provide physical mailing address of corresponding author	1
Contribution	#3b	Describe contributions of protocol authors and identify the guarantor of the review	13

Amendments

	#4	If the protocol represents an amendment of a previously completed or published protocol, identify as such and list changes; otherwise, state plan for documenting important protocol amendments	n/a

Support

Sources	#5a	Indicate sources of financial or other support for the review	13
Sponsor	#5b	Provide name for the review funder and / or sponsor	13
Role of sponsor or funder	#5c	Describe roles of funder(s), sponsor(s), and / or institution(s), if any, in developing the protocol	13

Introduction

For peer review only

Rationale	#6	Describe the rationale for the review in the context of what is already known	3-5
Objectives	#7	Provide an explicit statement of the question(s) the review will address with reference to participants, interventions, comparators, and outcomes (PICO)	5, 6
**Methods**			
Eligibility criteria	#8	Specify the study characteristics (such as PICO, study design, setting, time frame) and report characteristics (such as years considered, language, publication status) to be used as criteria for eligibility for the review	6-8
Information sources	#9	Describe all intended information sources (such as electronic databases, contact with study authors, trial registers or other grey literature sources) with planned dates of coverage	8
Search strategy	#10	Present draft of search strategy to be used for at least one electronic database, including planned limits, such that it could be repeated	8
Study records - data management	#11a	Describe the mechanism(s) that will be used to manage records and data throughout the review	8
Study records - selection process	#11b	State the process that will be used for selecting studies (such as two independent reviewers) through each phase of the review (that is, screening, eligibility and inclusion in meta-analysis)	8,9

For peer review only

Study records - data collection process	#11c	Describe planned method of extracting data from reports (such as piloting forms, done independently, in duplicate), any processes for obtaining and confirming data from investigators	9
Data items	#12	List and define all variables for which data will be sought (such as PICO items, funding sources), any pre-planned data assumptions and simplifications	9
Outcomes and prioritization	#13	List and define all outcomes for which data will be sought, including prioritization of main and additional outcomes, with rationale	7
Risk of bias in individual studies	#14	Describe anticipated methods for assessing risk of bias of individual studies, including whether this will be done at the outcome or study level, or both; state how this information will be used in data synthesis	9, 10
Data synthesis	#15a	Describe criteria under which study data will be quantitatively synthesised	10, 11
Data synthesis	#15b	If data are appropriate for quantitative synthesis, describe planned summary measures, methods of handling data and methods of combining data from studies, including any planned exploration of consistency (such as I2, Kendall's τ)	10, 11
Data synthesis	#15c	Describe any proposed additional analyses (such as sensitivity or subgroup analyses, meta-regression)	10, 11

For peer review only

Data synthesis	#15d	If quantitative synthesis is not appropriate, describe the type of summary planned	10,11
Meta-bias(es)	#16	Specify any planned assessment of meta-bias(es) (such as publication bias across studies, selective reporting within studies)	10,11
Confidence in cumulative evidence	#17	Describe how the strength of the body of evidence will be assessed (such as GRADE)	10

The PRISMA-P checklist is distributed under the terms of the Creative Commons Attribution License CC-BY 4.0. This checklist was completed on 20. November 2019 using https://www.goodreports.org/, a tool made by the EQUATOR Network in collaboration with Penelope.ai

For peer review only

BMJ Open

HEALTH SERVICE USE AMONG ADULTS WITH CEREBRAL PALSY: A MIXED METHODS SYSTEMATIC REVIEW PROTOCOL.

Journal:	*BMJ Open*
Manuscript ID	bmjopen-2019-035892.R2
Article Type:	Protocol
Date Submitted by the Author:	24-Apr-2020

Complete List of Authors:	Manikandan, Manjula; Royal College of Surgeons in Ireland, Department of Public Health and Epidemiology<br>Walsh, Aisling; Royal College of Surgeons in Ireland, Department of Public Health and Epidemiology<br>Kerr, C ; Queen's University Belfast, School of Nursing and Midwifery<br>Walsh, Michael; Health Service Executive, Office of the Chief Clinical Officer<br>Ryan, Jennifer; Brunel University London, College of Health and Life Sciences; Royal College of Surgeons in Ireland, Department of Public Health and Epidemiology

**Primary Subject Heading**:	Health services research
Secondary Subject Heading:	Neurology
Keywords:	Adult neurology < NEUROLOGY, REHABILITATION MEDICINE, HEALTH SERVICES ADMINISTRATION & MANAGEMENT

SCHOLARONE™
Manuscripts

I, the Submitting Author has the right to grant and does grant on behalf of all authors of the Work (as defined in the below author licence), an exclusive licence and/or a non-exclusive licence for contributions from authors who are: i) UK Crown employees; ii) where BMJ has agreed a CC-BY licence shall apply, and/or iii) in accordance with the terms applicable for US Federal Government officers or employees acting as part of their official duties; on a worldwide, perpetual, irrevocable, royalty-free basis to BMJ Publishing Group Ltd ("BMJ") its licensees and where the relevant Journal is co-owned by BMJ to the co-owners of the Journal, to publish the Work in this journal and any other BMJ products and to exploit all rights, as set out in our licence.

The Submitting Author accepts and understands that any supply made under these terms is made by BMJ to the Submitting Author unless you are acting as an employee on behalf of your employer or a postgraduate student of an affiliated institution which is paying any applicable article publishing charge ("APC") for Open Access articles. Where the Submitting Author wishes to make the Work available on an Open Access basis (and intends to pay the relevant APC), the terms of reuse of such Open Access shall be governed by a Creative Commons licence – details of these licences and which Creative Commons licence will apply to this Work are set out in our licence referred to above.

Other than as permitted in any relevant BMJ Author's Self Archiving Policies, I confirm this Work has not been accepted for publication elsewhere, is not being considered for publication elsewhere and does not duplicate material already published. I confirm all authors consent to publication of this Work and authorise the granting of this licence.

For peer review only

HEALTH SERVICE USE AMONG ADULTS WITH CEREBRAL PALSY: A MIXED METHODS SYSTEMATIC REVIEW PROTOCOL.

Manjula Manikandan[1], Aisling Walsh[1], Claire Kerr[2], Michael Walsh[4], Jennifer Ryan[1, 3]

[1]Department of Public Health and Epidemiology, Royal College of Surgeons in Ireland, Ireland

[2]School of Nursing and Midwifery, Queen's University Belfast, United Kingdom

[3]College of Health and Life Sciences, Brunel University London, United Kingdom

[4]Office of the Chief Clinical Officer, Health Service Executive, Ireland

Email: manjulamanikandan@rcsi.ie, aislingwalsh@rcsi.ie, c.kerr@qub.ac.uk, michael.walsh14@hse.ie, Jennifer.ryan@brunel.ac.uk / jenniferryan@rcsi.ie

Corresponding author: Jennifer Ryan, Mary Seacole Building, Brunel University London, Uxbridge, UB83PH, United Kingdom.

Telephone number of the corresponding author: +441895268702

Keywords: adults; cerebral palsy; health service use; experiences; protocol; systematic review

Word count: 2882 words (excluding references)

Abstract word count: 298 words

For peer review only - http://bmjopen.bmj.com/site/about/guidelines.xhtml

ABSTRACT

Introduction: Cerebral palsy (CP) is a neurodisability that primarily results in motor impairments and activity limitations, but is often associated with epilepsy and disturbances of sensation, perception, cognition, behaviour and speech. Most children with CP survive well into adulthood. Adults with CP experience increased risk of age-related chronic conditions such as arthritis, stroke, cardio-respiratory and mental health conditions in addition to the ongoing disabilities experienced from childhood. Therefore, adults with CP often require extensive health services. However, health service use among adults with CP has not been well documented. This mixed method review aims to identify, appraise and synthesise quantitative and qualitative literature examining health service use among adults with CP.

Methods and analysis: The mixed method systematic review will be conducted in accordance with the Joanna Briggs Institute (JBI) methodology. A systematic search of Medline (Ovid), CINAHL, EMBASE, PsychINFO and Cochrane library from inception to March 2020 will be conducted. Quantitative observational studies, qualitative studies, and mixed method studies examining health service use among adults with CP (≥ 18 years) will be included. Outcomes of interest are the proportion of adults using health services frequency of use and experiences of health services from the perspectives of adults with CP, caregivers and health service providers. . Two reviewers will independently screen titles, abstracts and full texts, extract data, and assess the quality of included studies using JBI instruments. Where possible a pooled analysis and aggregation of findings will be performed for quantitative and qualitative data, respectively, and GRADE/GRADE-CerQual employed. Quantitative and qualitative findings will be integrated using a triangulation approach at the synthesis stage. A narrative synthesis will be carried out where this is not possible.

Ethics and dissemination: Ethical approval is not required for this review. The findings will be disseminated through a peer-reviewed journal and conferences.

For peer review only - http://bmjopen.bmj.com/site/about/guidelines.xhtml

Systematic review registration number: The Prospero registration number is 155380.

STRENGTHS & LIMITATIONS OF THIS STUDY

- This mixed method systematic review will include quantitative, qualitative and mixed methods studies in order to provide a comprehensive overview of the current literature relating to health services for adults with cerebral palsy.
- This review process is strengthened by utilisation of the widely used Andersen-Newman behavioural model of health service utilisation as an overarching framework to guide data extraction and analysis.
- Only studies that are published in English will be included in the review.
- Heterogeneity of eligible studies may limit the possibility of meta-analysis.

BACKGROUND

Cerebral palsy (CP) is a "group of permanent disorders of movement and posture, causing activity limitation, that are linked to non-progressive disturbances of the developing fetal or infant brain" [1].The disturbance to the brain can occur before, during or up to approximately 2 years after birth from traumatic or non-traumatic brain injury [2 3] . With recent medical advances, CP can be diagnosed accurately before the age of 6 months [4]. People with CP present with different types of motor abnormality, namely spasticity, dyskinesia and ataxia [1 5]. In addition to movement disorders, people with CP may experience epilepsy or impairments of cognition, speech, hearing and vision [1 5].

Traditionally, CP was considered a paediatric condition. The incidence rate of CP is approximately between 2.0-2.5 cases per 1000 live births wordwide [6-9]. However recently the birth prevalence has decreased in settings including Europe and Australia [6-9]. Many people with CP survive well into adulthood with a recent study reporting more than 80% of people with CP have a life expectancy, beyond 58 years [10]. People with

For peer review only - http://bmjopen.bmj.com/site/about/guidelines.xhtml

CP continue to present with ongoing health issues from childhood, in addition to the issues related to ageing. For instance, adults with CP experience increased risk of arthritis, stroke, heart failure, ischaemic heart disease, chronic respiratory disease and mental health disorders compared to adults without CP [11-14]. Studies have also reported increased fatigue, incidence of falls, pain and a decline in mobility, as people with CP move into adulthood [15-17]. Therefore, adults with CP may require extensive health services.

The National Institute for Health and Care Excellence (NICE) guideline for CP in adults has recommended continuity of care for adults with CP, tailored to their needs [18]. This continuity of care is likely to avoid emergency hospital admissions[18]. However, studies of adolescents with CP have reported challenging experiences in navigating services during transition from child to adult services [19 20]. In addition, adolescents with CP reported anxiety about transfer from child to adult services as they perceived that services in adulthood were poorly co-ordinated and managed [19-21]. As the evidence describing the long-term health issues experienced by adults with CP has expanded in recent years, there is a growing need for research examining health service use among adults with CP.

'Health services' are defined as those "services that are first and foremost undertaken to have direct effect on people's health. These extend from health promotion and disease prevention, through curative services, to long term care and rehabilitation"[22]. Use of health services may be associated with various factors relating to the individual and their environment [23 24]. The previous Andersen-Newman behavioural models of health service utilisation (ANM) proposed family and individual as the unit of analysis for health service use [24]. The revised Andersen-Newman behavioural model of health service utilisation (ANM) provides a framework for considering health service use [23]. It describes environmental factors, population factors and outcomes that can be associated with health service use behaviour [23]. Environmental factors

For peer review only - http://bmjopen.bmj.com/site/about/guidelines.xhtml

include those relating to the health care system (e.g., national policies, resources and organisations involved) and external environment (e.g., physical, political and economic factors) [23]. Population factors include demographics (e.g. age, gender, ethnicity, location and condition-specific characteristics such as functional mobility and type of motor abnormality) [23]. Outcomes include perceived and evaluated health status and customer satisfaction [23].Over the years, ANM has been adapted, critiqued and reviewed extensively [25-29]. However, the revised ANM (1995) includes environment as a key driver of the model impacting health service use behaviour [23]. It also emphasizes the relationship between factors through feedback loops, which demonstrates the complexity involved in health service use behaviour [23] .

A preliminary search of PROSPERO, MEDLINE (Ovid), JBI database and the Cochrane Database of Systematic Reviews indicated that no systematic reviews have been conducted examining health service use among adults with CP. Previous reviews have focused on the epidemiology of CP in adults, quality of life, pain and effectiveness of interventions used by adults with CP[16 30-32]. There is a need for a theory informed review with broader perspectives on health service use among adults with CP, rather than focusing on the effectiveness of any specific intervention. Such a review would potentially inform health service used by adults with CP, direct research effortsand identify areas of challenges faced by adults with CP. Therefore, the aim of this mixed method systematic review is to identify, appraise and synthesise the available quantitative and qualitative literature examining health service use among adults with CP using the ANM as a framework.

OBJECTIVES

The objectives are:

1. To determine the proportion and frequency of health service use by adults with CP.
2. To examine associations between environmental factors, population factors, outcomes and health service use.

For peer review only - http://bmjopen.bmj.com/site/about/guidelines.xhtml

3. To explore the experiences and perceptions of health service use for adults with CP, from the perspective of adults with CP, caregivers and health service providers.

METHODS

This mixed method systematic review will be conducted in accordance with the Joanna Briggs Institute methodology guidelines [33]. The protocol was submitted for registration on the PROSPERO database of systematic reviews [receipt number: 155380]. This review is written according to the PRISMA-P checklist [34]. The review will be reported using MOOSE and ENTREQ reporting guidelines [35 36].

Study eligibility

Study design

The review will include quantitative, qualitative and mixed method studies. Quantitative studies will include observational studies (e.g., cross-sectional, case-control and cohort studies). Qualitative studies will include studies of recognised qualitative approaches (e.g., interviews, focus groups, observation, thematic analysis, content analysis, narrative analysis and framework analysis) [37]. Mixed method studies will only be considered if quantitative or qualitative components can be clearly extracted.

Studies published in English language will be included. There will be no restriction on the geographic location of studies or date of publication

Participants

Studies of adults with CP (≥ 18 years) with all types of motor disorders and all levels of functional mobility will be included for both quantitative and qualitative studies. Functional mobility for people with CP is widely classified using the Gross Motor Function Classification System (GMFCS) [38]. For qualitative studies, articles including caregivers (also includes family members) and health service providers will also be included. For studies that include children and young adults (for example, those aged 15-25 years), data

For peer review only - http://bmjopen.bmj.com/site/about/guidelines.xhtml

For peer review only

from people ≥18 years only will be included, if possible. If it is not possible to extract data for adults only, we will include data if the mean age of the sample is ≥18 years. Where studies include data on adults with CP as part of a larger sample of people with disabilities, we will extract data on adults with CP only, if possible.

Outcome

Health services for this review will include services that have direct impact on the health of adults with CP [22 39-42]. This can include hospital admissions, emergency department visits, outpatient visits to medical and allied health professionals (e.g. physicians, pharmacist, physiotherapists, occupational therapists, podiatrists, psychologists, orthotists, speech and language therapists). In addition, diagnostic or assistive device services (e.g. radiologists, communication aids, mobility aids and wheelchair services) and support services (e.g. supported living, personal assistance, residential, respite and domiciliary services) will also be included [22 39-42].

For the quantitative component of this review, the proportion of adults using health services and the frequency of health service use will be the main outcomes. Where possible, we will report each type of health services used by adults with CP.

For the qualitative component, the main outcomes are experience and perceptions of using health services among adults with CP. We will also explore experiences and perceptions of health services for adults with CP from the perspectives of caregivers, and health service providers.

Exclusion criteria

We will exclude reviews, dissertations, editorials, commentaries and conference abstracts. Studies of children and young people with CP (<18 years) will be excluded. Studies examining services that have an indirect effect on people's health only such as education, employment, housing and transport will be

For peer review only - http://bmjopen.bmj.com/site/about/guidelines.xhtml

excluded [22]. Finally, randomised controlled studies on intervention effectiveness, pragmatic, open-label trials, case series and case reports will be excluded from this review.

Search Strategy

The electronic databases Medline (Ovid), CINAHL, EMBASE, PsychINFO and Cochrane library will be systematically searched from inception of each database to March 2020. We will conduct a pilot search in Medline (Ovid) to inform the search strategy. A librarian will support development of the search strategy. Reference lists of included studies will be screened.

Search terms

The search strategy will consist of fully exploded subject headings and free text words relating to adults with CP and health services. The search strategy developed from Medline (Ovid) will be tailored to each database. No filters on study design or settings will be applied at this stage. Search terms will be combined using Boolean operators. A sample search strategy for Medline (Ovid) is provided in Appendix 1.

Data management

A search log will be maintained to record the databases, keywords used and results of each search. The titles and abstracts of papers identified from the systematic search will be imported into reference management software (Endnote X8.2) and duplicates will be removed.

Study selection

Two reviewers will screen titles and abstracts independently using Rayyan software [43]. Where studies meet the inclusion criteria or it is unclear if they are eligible, full-texts will be retrieved for further assessment. The two reviewers will screen full text articles for inclusion independently. Conflicts will be resolved by discussion or third reviewer involvement. The reasons for exclusion of full text studies will be

For peer review only - http://bmjopen.bmj.com/site/about/guidelines.xhtml

recorded and the summary of the search results will be presented in a Preferred Reporting Items for Systematic Reviews and Meta-analyses (PRISMA) flow diagram [44].

Data extraction

Two reviewers will extract data independently using the JBI data extraction tool for quantitative and qualitative studies accordingly. Any disagreements will be resolved initially through discussion and if required, consultation with a third reviewer. The study authors will be contacted to retrieve missing data necessary for meta-analysis or meta-aggregation.

Data items

For quantitative studies and quantitative components of mixed method studies, extracted data will include study details, study design, country, funding sources, declaration of interests, participant characteristics, exposure and outcome of interest [45]. This will include proportion of people using each health service, frequency of use of each health service, and variables relating to the environmental factors, population factors and outcomes according to the ANM. For qualitative studies and the qualitative components of mixed method studies, the extracted data will include study design, setting, country, methodology, funding sources, declaration of interests, participants, data analysis and themes related to experiences [46]. The qualitative data, such as quotes or themes of experiences and perceptions will be extracted from the results sections of the included studies.

Assessment of methodological quality

Two reviewers will independently assess the methodological quality of included primary studies using standardised critical appraisal instruments from the Joanna Briggs Institute[33]. For quantitative studies the

For peer review only - http://bmjopen.bmj.com/site/about/guidelines.xhtml

appropriate JBI- critical appraisal checklist for cohort, case-control and analytical cross-sectional studies will be used [45]. For qualitative studies the JBI- qualitative appraisal instrument will be used [46]. Quality appraisal will be undertaken at study level and studies will not be excluded for data extraction or synthesis based on the methodological quality assessment, as this review seeks to capture all experiences of health service use by people with CP, and so necessitates inclusion of studies of lower methodological quality. Disagreements between the reviewers will be discussed and discrepancies resolved by a third reviewer. The results of the quality appraisal will be reported in a table and narrative form.

Data synthesis

Quantitative studies

The pooled proportion of people using each type of health services and pooled mean visits to each type of health service will be estimated using a random effects model. We will standardise frequency to a specific time frame. Separate meta-analyses will be conducted for each service. We will use random-effects model due to the likely heterogeneity of the population and outcome variable [47]. We will assess statistical heterogeneity using the I^2 statistic and a Chi^2 test [48]. If studies compare health service use between people with and without CP, we will calculate an odds ratio for dichotomous data and a standardized mean difference for continuous data. If data allow, we will conduct separate analysis for high and Low or Middle Income Countries (LMIC) as defined by the World Bank Countries classification to examine regional or income based variations in health service use among adults with CP [49-51]. Where data cannot be pooled, a narrative synthesis of the quantitative findings will be conducted.

Qualitative studies

Qualitative experiences from different perspectives will be pooled using a meta-aggregation approach [52]. This involves identifying the findings, categorising the findings based on similarity in meaning and

For peer review only - http://bmjopen.bmj.com/site/about/guidelines.xhtml

grouping the categories into synthesised findings [52]. Where textual-pooling is not possible, the findings will be presented in narrative form.

Integration

The findings from quantitative and qualitative synthesis will be integrated using a convergent segregated approach, to preserve the integrity of quantitative and qualitative findings [33]. The results from quantitative and qualitative components will be triangulated and reported using the ANM as a framework. Triangulation will involve comparing or organising quantitative and qualitative findings into a line of argument via tabulation, groupings and visual representation to produce an overall configured analysis [53]. A narrative form of discussion will be considered, where this is not possible.

Confidence in cumulative evidence

GRADE will be used to interpret the quantitative review findings and demonstrate the overall quality of the data [54]. GRADE-CERQual will be used to interpret the qualitative review findings in the context of methodological limitations, relevance, coherence and adequacy of data [55]. According to the JBI guidelines for mixed methods reviews, there are complexities involved in assessing confidence in findings from combined quantitative and qualitative evidence [33]. Therefore, the GRADE approach will only be used for separate qualitative and quantitative evidence analysis, but not for assessing the integrated findings

ETHICS AND DISSEMINATION

Ethical approval is not required for this review as both quantitative and qualitative data used for analysis will be extracted from published studies. The review will be published in a peer-reviewed journal and presented at conferences for researchers, health professionals and people with CP. Findings will be disseminated to healthcare providers and shared with charity and voluntary agencies that support people with CP.

For peer review only - http://bmjopen.bmj.com/site/about/guidelines.xhtml

Patient and Public Involvement (PPI)

Adults with CP and professionals will be involved in interpretation and dissemination of the review findings.

DISCUSSION

This mixed methods review will provide greater insights of the available literature on the health services used by adults with CP and related experiences of health services from the perspectives of adults with CP, their caregivers and health service providers. The majority of people with CP will survive to at least 60 years of age [10]. Many adults with CP experience secondary conditions with age that require intervention and support from clinicians working across multiple health and social care disciplines[12]. Previous research has demonstrated that young people with CP experience challenges in accessing health services, as they move from child to adult health services [19 21 56-58]. Therefore, a review of health service use among adults with CP is required in order to direct research efforts and inform service provision.

A theory-informed review using the revised ANM will guide examination of factors associated with health service use among adults with CP[23]. This model describes factors by environment, population and outcomes influencing health service use behaviour with feedback loops showing the complexities involved[23]. However, the ANM provides only a cross-sectional view on health service use and does not include change or life course as a factor [29]. This incomplete view of a phenomenon is common among most theoretical frameworks used in the literature [59]. The life course perspective provides a complete understanding of past versus present service use and the changes seen over time[29]. Where possible a narrative synthesis will be done for life course or change over time in service use.

For peer review only - http://bmjopen.bmj.com/site/about/guidelines.xhtml

A limitation of this review is the exclusion of studies that are not peer-reviewed or published in languages other than English, potentially resulting in less generalisable findings from other languages. Finally, it may not be possible to conduct a meta-analysis due to a lack of / and/or heterogeneity of data.

AUTHOR CONTRIBUTIONS

All authors have agreed on the final version and meet at least two of the following criteria (recommended by ICMJE www.icmje.org/recommendations):

1. Substantial contributions to the conception or design of the work; or the acquisition, analysis, or interpretation of data for the work **(MM, AW, CK, MW and JR)**
2. Drafting the work or revising it critically for important intellectual content (**MM, JR)**
3. Final approval of the version to be published **(MM, AW, CK, MW and JR)**
4. Agreement to be accountable for all aspects of the work in ensuring that questions related to the accuracy or integrity of any part of the work are appropriately investigated and resolved **(MM and JR).**

ACKNOWLEDGEMENTS

The authors acknowledge the support from the RCSI librarian (Ms Grainne McCabe) for supporting the development of search terms with the reviewers.

COMPETING INTERESTS

None declared.

FUNDING

This work was conducted as part of the SPHeRE Programme under Grant No. SPHeRE/2013/1. This review is funded by the Royal College of Surgeons in Ireland (RCSI) through the StAR programme. The funders had no role in developing the protocol.

For peer review only - http://bmjopen.bmj.com/site/about/guidelines.xhtml

REFERENCES

1. Colver A, Fairhurst C, Pharoah PO. Cerebral palsy. *Lancet (London, England)* 2014;383(9924):1240-49. doi: 10.1016/s0140-6736(13)61835-8 [published Online First: 2013/11/26]

2. McIntyre S, Morgan C, Walker K, et al. Cerebral Palsy—Don't Delay. *Developmental Disabilities Research Reviews* 2011;17(2):114-29. doi: 10.1002/ddrr.1106

3. Granild-Jensen JB, Rackauskaite G, Flachs EM, et al. Predictors for early diagnosis of cerebral palsy from national registry data. *Developmental Medicine & Child Neurology* 2015;57(10):931-35. doi: 10.1111/dmcn.12760

4. Novak I, Morgan C, Adde L, et al. Early, Accurate Diagnosis and Early Intervention in Cerebral Palsy: Advances in Diagnosis and Treatment. *JAMA Pediatrics* 2017;171(9):897-907. doi: 10.1001/jamapediatrics.2017.1689 [published Online First: 2017/07/18]

5. Novak I, Hines M, Goldsmith S, et al. Clinical prognostic messages from a systematic review on cerebral palsy. *Pediatrics* 2012;130(5):e1285-312. doi: 10.1542/peds.2012-0924 [published Online First: 2012/10/10]

6. Johnson A. Prevalence and characteristics of children with cerebral palsy in Europe. *Developmental Medicine & Child Neurology* 2002;44(9):633-40. doi: 10.1017/S0012162201002675 [published Online First: 2002/09/16]

7. Oskoui M, Coutinho F, Dykeman J, et al. An update on the prevalence of cerebral palsy: a systematic review and meta-analysis. *Developmental Medicine & Child Neurology* 2013;55(6):509-19. doi: 10.1111/dmcn.12080 [published Online First: 2013/01/26]

8. Galea C, Mcintyre S, Smithers-Sheedy H, et al. Cerebral palsy trends in Australia (1995–2009): a population-based observational study. *Developmental Medicine & Child Neurology* 2019;61(2):186-93. doi: 10.1111/dmcn.14011

9. Sellier E, Platt MJ, Andersen GL, et al. Decreasing prevalence in cerebral palsy: a multi-site European population-based study, 1980 to 2003. *Developmental Medicine & Child Neurology* 2016;58(1):85-92. doi: 10.1111/dmcn.12865

10. Blair E, Langdon K, McIntyre S, et al. Survival and mortality in cerebral palsy: observations to the sixth decade from a data linkage study of a total population register and National Death Index. *BMC Neurology* 2019;19(1):111.

For peer review only - http://bmjopen.bmj.com/site/about/guidelines.xhtml

For peer review only

11. Peterson MD, Ryan JM, Hurvitz EA, et al. Chronic Conditions in Adults With Cerebral Palsy. *JAMA* 2015;314(21):2303-5. doi: 10.1001/jama.2015.11025 [published Online First: 2015/12/02]

12. Ryan JM, Peterson MD, Matthews A, et al. Noncommunicable disease among adults with cerebral palsy: A matched cohort study. *Neurology* 2019;93(14):e1385-e96.

13. Smith KJ, Peterson MD, O'Connell NE, et al. Risk of depression and anxiety in adults with cerebral palsy. *JAMA Neurology* 2019;76(3):294-300.

14. O'Connell NE, Smith KJ, Peterson MD, et al. Incidence of osteoarthritis, osteoporosis and inflammatory musculoskeletal diseases in adults with cerebral palsy: A population-based cohort study. *Bone* 2019;125:30-35.

15. Bottos M, Feliciangeli A, Sciuto L, et al. Functional status of adults with cerebral palsy and implications for treatment of children. *Developmental Medicine & Child Neurology* 2001;43(8):516-28.

16. van Gorp M, Hilberink SR, Noten S, et al. The epidemiology of cerebral palsy in adulthood: A systematic review and meta-analysis of the most frequently studied outcomes. *Archives of Physical Medicine and Rehabilitation* 2020

17. Ryan JM, Cameron MH, Liverani S, et al. Incidence of falls among adults with cerebral palsy: a cohort study using primary care data. *Developmental Medicine & Child Neurology* 2020;62(4):477-82. doi: 10.1111/dmcn.14444 [published Online First: 2019/12/28]

18. National Institute for Health and Care Excellence (NICE). Cerebral Palsy in adults London 2019 [Available from: https://www.nice.org.uk/guidance/ng119/chapter/Rationale-and-impact.

19. Hislop J, Mason H, Parr JR, et al. Views of Young People With Chronic Conditions on Transition From Pediatric to Adult Health Services. *Journal of Adolescent Health* 2016;59(3):345-53. doi: https://doi.org/10.1016/j.jadohealth.2016.04.004

20. Wright AE, Robb J, Shearer MC. Transition from paediatric to adult health services in Scotland for young people with cerebral palsy. *Journal of Child Health Care : for professionals working with children in the hospital and community* 2016;20(2):205-13. doi: 10.1177/1367493514564632 [published Online First: 2015/01/17]

21. Colver A, Pearse R, Watson RM, et al. How well do services for young people with long term conditions deliver features proposed to improve transition? *BMC Health Service Research* 2018;18(1):337. doi: 10.1186/s12913-018-3168-9 [published Online First: 2018/05/10]

For peer review only - http://bmjopen.bmj.com/site/about/guidelines.xhtml

22. Black N, Gruen R. Understanding health services: McGraw-Hill Education (UK) 2005.

23. Andersen RM. Revisiting the behavioral model and access to medical care: does it matter? *Journal of Health and Social Behavior* 1995:1-10.

24. Andersen R, Newman JF. Societal and individual determinants of medical care utilization in the United States. *The Milbank Memorial Fund Quarterly Health and Society* 1973:95-124.

25. Goldsmith LJ. Access to health care for disadvantaged individuals: A qualitative inquiry. 2007

26. Babitsch B, Gohl D, von Lengerke T. Re-revisiting Andersen's Behavioral Model of Health Services Use: a systematic review of studies from 1998-2011. *Psychosoc Med* 2012;9:Doc11-Doc11. doi: 10.3205/psm000089 [published Online First: 2012/10/25]

27. Guilcher SJ, Craven BC, McColl MA, et al. Application of the Andersen's health care utilization framework to secondary complications of spinal cord injury: a scoping review. *Disability and Rehabilitation* 2012;34(7):531-41.

28. Phillips KA, Morrison KR, Andersen R, et al. Understanding the context of healthcare utilization: assessing environmental and provider-related variables in the behavioral model of utilization. *Health Services Research* 1998;33(3 Pt 1):571.

29. O'Donovan M-A, McCallion P, McCarron M, et al. A narrative synthesis scoping review of life course domains within health service utilisation frameworks. *HRB Open Research* 2019;2(6):6.

30. Alves-Nogueira A, Silva N, McConachie H, et al. A systematic review on quality of life assessment in adults with cerebral palsy: Challenging issues and a call for research. *Research in Developmental Disabilities* 2020;96:103514.

31. van der Slot WM, Benner JL, Brunton L, et al. Pain in adults with cerebral palsy: a systematic review and meta-analysis of individual participant data. *Annals of Physical and Rehabilitation Medicine* 2020

32. Adams CT, Lakra A. Clinical and Functional Outcomes of Total Hip Arthroplasty in Patients with Cerebral Palsy: A Systematic Review. *Journal of Orthopaedics* 2020

33. Lizarondo L SC, Carrier J, Godfrey C, Rieger K, Salmond S, Apostolo J, Kirkpatrick P, Loveday, Hadders-Algra, M. Chapter 8: Mixed methods systematic reviews. In: Aromataris E, Munn Z (Editors). Joanna Briggs Institute Reviewer's Manual. The Joanna Briggs Institute 2017 [Available from: https://reviewersmanual.joannabriggs.org/.

For peer review only - http://bmjopen.bmj.com/site/about/guidelines.xhtml

34. Moher D, Shamseer L, Clarke M, et al. Preferred reporting items for systematic review and meta-analysis protocols (PRISMA-P) 2015 statement. *Systematic Reviews* 2015;4(1):1.

35. Tong A, Flemming K, McInnes E, et al. Enhancing transparency in reporting the synthesis of qualitative research: ENTREQ. *BMC Medical Research Methodology* 2012;12:181. doi: 10.1186/1471-2288-12-181 [published Online First: 2012/11/29]

36. Stroup DF, Berlin JA, Morton SC, et al. Meta-analysis of observational studies in epidemiology: a proposal for reporting. Meta-analysis Of Observational Studies in Epidemiology (MOOSE) group. *JAMA* 2000;283(15):2008-12. [published Online First: 2000/05/02]

37. Creswell JW, Poth CN. Qualitative inquiry and research design: Choosing among five approaches. 4th ed: Sage Publications 2017.

38. Palisano R, Rosenbaum P, Walter S, et al. Development and reliability of a system to classify gross motor function in children with cerebral palsy. *Developmental Medicine and Child Neurology* 1997;39(4):214-23. doi: 10.1111/j.1469-8749.1997.tb07414.x [published Online First: 1997/04/01]

39. Cheng SM, Catallo C. Case definition for health and social care services integrated initiatives. *Journal of Integrated Care* 2019

40. Leutz WN. Five laws for integrating medical and social services: lessons from the United States and the United Kingdom. *The Milbank Quarterly* 1999;77(1):77-110.

41. Leichsenring K. Developing integrated health and social care services for older persons in Europe. *International Journal of Integrated Care* 2004;4:e10-e10. doi: 10.5334/ijic.107

42. Dickens J. The definition of social work in the United Kingdom, 2000–2010. *International Journal of Social Welfare* 2012;21(1):34-43. doi: 10.1111/j.1468-2397.2011.00782.x

43. Ouzzani M, Hammady H, Fedorowicz Z, et al. Rayyan-a web and mobile app for systematic reviews. *Systematic Reviews* 2016;5(1):210-10. doi: 10.1186/s13643-016-0384-4

44. Moher D, Liberati A, Tetzlaff J, et al. Preferred reporting items for systematic reviews and meta-analyses: the PRISMA statement. *Annals of Internal Medicine* 2009;151(4):264-69.

45. Moola S MZ, Tufanaru C, Aromataris E, Sears K, Sfetcu R, Currie M, Lisy K, Qureshi R, Mattis P, Mu P. Chapter 7: Systematic reviews of etiology and risk. In: Aromataris E, Munn Z (Editors). Joanna

For peer review only - http://bmjopen.bmj.com/site/about/guidelines.xhtml

Briggs Institute Reviewer's Manual. The Joanna Briggs Institute. 2017 [Available from: https://reviewersmanual.joannabriggs.org/

46. Lockwood C PK, Munn Z, Rittenmeyer L, Salmond S, Bjerrum M, Loveday H, Carrier J, Stannard D. Chapter 2: Systematic reviews of qualitative evidence. In: Aromataris E, Munn Z (Editors). Joanna Briggs Institute Reviewer's Manual. The Joanna Briggs Institute. 2017 [Available from: https://reviewersmanual.joannabriggs.org/.

47. Borenstein M, Hedges LV, Higgins JPT, et al. A basic introduction to fixed-effect and random-effects models for meta-analysis. *Research Synthesis Methods* 2010;1(2):97-111. doi: 10.1002/jrsm.12

48. Munn Z, Tufanaru C, Aromataris E. JBI's systematic reviews: data extraction and synthesis. *AJN The American Journal of Nursing* 2014;114(7):49-54.

49. World Bank. World Bank Country and Lending Groups 2019 [Available from: https://datahelpdesk.worldbank.org/knowledgebase/articles/906519-world-bank-country-and-lending-groups accessed 17 April 2020.

50. World Bank. How does the World Bank classify countries? [Available from: https://datahelpdesk.worldbank.org/knowledgebase/articles/378834-how-does-the-world-bank-classify-countries accessed 17 April 2020.

51. Mills A, Hsu J. Health Services in Low- and Middle-Income Countries: Financing, Payment, and Provision. In: Anthony J. Culyer (ed.), Encyclopedia of Health Economics2014:422-34.

52. Pearson A. Balancing the evidence: incorporating the synthesis of qualitative data into systematic reviews. *JBI reports* 2004;2(2):45-64.

53. Noyes J, Booth A, Moore G, et al. Synthesising quantitative and qualitative evidence to inform guidelines on complex interventions: clarifying the purposes, designs and outlining some methods. *BMJ Global Health* 2019;4(Suppl 1):e000893.

54. GRADE. Grading of Recommendations Assessment, Development and Evaluation GRADE working group [Available from: https://www.gradeworkinggroup.org/ accessed 03 March 2020.

55. Lewin S, Glenton C, Munthe-Kaas H, et al. Using qualitative evidence in decision making for health and social interventions: an approach to assess confidence in findings from qualitative evidence syntheses (GRADE-CERQual). *PLOS Medicine* 2015;12(10)

For peer review only

For peer review only - http://bmjopen.bmj.com/site/about/guidelines.xhtml

56. Colver A, McConachie H, Le Couteur A, et al. A longitudinal, observational study of the features of transitional healthcare associated with better outcomes for young people with long-term conditions. *BMC Medicine* 2018;16(1):111.

57. Bagatell N, Chan D, Rauch KK, et al. "Thrust into adulthood": Transition experiences of young adults with cerebral palsy. *Disability and Health Journal* 2017;10(1):80-86. doi: https://doi.org/10.1016/j.dhjo.2016.09.008

58. Kolehmainen N, McCafferty S, Maniatopoulos G, et al. What constitutes successful commissioning of transition from children's to adults' services for young people with long-term conditions and what are the challenges? An interview study. *BMJ Paediatrics Open* 2017;1(1) doi: 10.1136/bmjpo-2017-000085

59. Shannon-Baker P. Making paradigms meaningful in mixed methods research. *Journal of Mixed Methods Research* 2016;10(4):319-34.

For peer review only

For peer review only - http://bmjopen.bmj.com/site/about/guidelines.xhtml

Appendix 1

Medline (Ovid)

Ovid MEDLINE(R) and Epub Ahead of Print, In-Process & Other Non-Indexed Citations, Daily and Versions(R) 1946 to present

1. exp Cerebral Palsy/
2. cerebral pals$.tw.
3. ((Hemiplegi$ or diplegi$ or quadriplegi$ or unilateral$) adj5 spastic$).tw.
4. ((Hemiplegi$ or diplegi$ or quadriplegi$ or unilateral$) adj3 ataxi$).tw.
5. Little$ disease.tw.
6. 1 or 2 or 3 or 4 or 5
7. exp Health Services/
8. exp Health Facilities/
9. exp Comprehensive Health Care/
10. exp "Delivery of Health Care"/
11. exp "analytical, diagnostic and therapeutic techniques and equipment (non mesh)"/
12. exp residential treatment/
13. emergenc$.tw.
14. therap$.tw.
15. diagnos$.tw.
16. assistive device$.tw.

For peer review only

17. health service$.tw.

18. health care$.tw.

19. hospital$.tw.

20. rehabilitation$.tw.

21. general practice$.tw.

22. family practice$.tw.

23. primary care$.tw.

24. community$.tw.

25. respite$.tw.

26. residential care$.tw

27. 7 or 8 or 9 or 10 or 11 or 12 or 13 or 14 or 15 or 16 or 17 or 18 or 19 or 20 or 21 or 22 or 23 or 24 or 25 or 26

28. exp Adult/

29. adult$.tw.

30. elder$.tw.

31. older$.tw.

32. young adult$.tw.

33. 28 or 29 or 30 or 31 or 32

34. 6 AND 27 AND 33

For peer review only

Reporting checklist for protocol of a systematic review.

Based on the PRISMA-P guidelines.

Instructions to authors

Complete this checklist by entering the page numbers from your manuscript where readers will find each of the items listed below.

Your article may not currently address all the items on the checklist. Please modify your text to include the missing information. If you are certain that an item does not apply, please write "n/a" and provide a short explanation.

Upload your completed checklist as an extra file when you submit to a journal.

In your methods section, say that you used the PRISMA-Preporting guidelines, and cite them as:

Moher D, Shamseer L, Clarke M, Ghersi D, Liberati A, Petticrew M, Shekelle P, Stewart LA. Preferred Reporting Items for Systematic Review and Meta-Analysis Protocols (PRISMA-P) 2015 statement. Syst Rev. 2015;4(1):1.

		Reporting Item	Page Number
**Title**			
Identification	#1a	Identify the report as a protocol of a systematic review	1
Update	#1b	If the protocol is for an update of a previous systematic review, identify as such	n/a

For peer review only

**Registration**			
	#2	If registered, provide the name of the registry (such as PROSPERO) and registration number	3
**Authors**			
Contact	#3a	Provide name, institutional affiliation, e-mail address of all protocol authors; provide physical mailing address of corresponding author	1
Contribution	#3b	Describe contributions of protocol authors and identify the guarantor of the review	13
**Amendments**			
	#4	If the protocol represents an amendment of a previously completed or published protocol, identify as such and list changes; otherwise, state plan for documenting important protocol amendments	n/a
**Support**			
Sources	#5a	Indicate sources of financial or other support for the review	13
Sponsor	#5b	Provide name for the review funder and / or sponsor	13
Role of sponsor or funder	#5c	Describe roles of funder(s), sponsor(s), and / or institution(s), if any, in developing the protocol	13
**Introduction**			

For peer review only

Rationale	#6	Describe the rationale for the review in the context of what is already known	3-5
Objectives	#7	Provide an explicit statement of the question(s) the review will address with reference to participants, interventions, comparators, and outcomes (PICO)	5, 6
**Methods**			
Eligibility criteria	#8	Specify the study characteristics (such as PICO, study design, setting, time frame) and report characteristics (such as years considered, language, publication status) to be used as criteria for eligibility for the review	6-8
Information sources	#9	Describe all intended information sources (such as electronic databases, contact with study authors, trial registers or other grey literature sources) with planned dates of coverage	8
Search strategy	#10	Present draft of search strategy to be used for at least one electronic database, including planned limits, such that it could be repeated	8
Study records - data management	#11a	Describe the mechanism(s) that will be used to manage records and data throughout the review	8
Study records - selection process	#11b	State the process that will be used for selecting studies (such as two independent reviewers) through each phase of the review (that is, screening, eligibility and inclusion in meta-analysis)	8,9

For peer review only

Study records - data collection process	#11c	Describe planned method of extracting data from reports (such as piloting forms, done independently, in duplicate), any processes for obtaining and confirming data from investigators	9
Data items	#12	List and define all variables for which data will be sought (such as PICO items, funding sources), any pre-planned data assumptions and simplifications	9
Outcomes and prioritization	#13	List and define all outcomes for which data will be sought, including prioritization of main and additional outcomes, with rationale	7
Risk of bias in individual studies	#14	Describe anticipated methods for assessing risk of bias of individual studies, including whether this will be done at the outcome or study level, or both; state how this information will be used in data synthesis	9, 10
Data synthesis	#15a	Describe criteria under which study data will be quantitatively synthesised	10, 11
Data synthesis	#15b	If data are appropriate for quantitative synthesis, describe planned summary measures, methods of handling data and methods of combining data from studies, including any planned exploration of consistency (such as I2, Kendall's τ)	10, 11
Data synthesis	#15c	Describe any proposed additional analyses (such as sensitivity or subgroup analyses, meta-regression)	10, 11

For peer review only

Data synthesis	#15d	If quantitative synthesis is not appropriate, describe the type of summary planned	10,11
Meta-bias(es)	#16	Specify any planned assessment of meta-bias(es) (such as publication bias across studies, selective reporting within studies)	10,11
Confidence in cumulative evidence	#17	Describe how the strength of the body of evidence will be assessed (such as GRADE)	11

The PRISMA-P checklist is distributed under the terms of the Creative Commons Attribution License CC-BY 4.0. This checklist was completed on 20. November 2019 using https://www.goodreports.org/, a tool made by the EQUATOR Network in collaboration with Penelope.ai

For peer review only
